# Inactivation of MST1/2 Controls Macrophage Polarization to Affect Macrophage-Related Disease via YAP and Non-YAP Mechanisms

**DOI:** 10.7150/ijbs.87057

**Published:** 2024-01-12

**Authors:** Yina An, Shuyu Tan, Pu Zhang, Jingjing Yang, Kezhi Wang, Ruicheng Zheng, Lu Qiao, Yang Wang, Yanjun Dong

**Affiliations:** 1 Department of Basic Veterinary Medicine, College of Veterinary Medicine, China Agricultural University; Beijing, 100193, China.; 2 Beijing Key Laboratory of Detection Technology for Animal-Derived Food Safety, College of Veterinary Medicine, China Agricultural University; Beijing, 100193, China.

**Keywords:** MST1/2, macrophage polarization, inflammatory disease, bacterial infection, UUO

## Abstract

Macrophage polarization is a critical process that regulates in inflammation, pathogen defense, and tissue repair. Here we demonstrate that MST1/2, a core kinase of Hippo pathway and a recently identified regulator of inflammation, plays a significant role in promoting M2 polarization. We provide evidence that inhibition of MST1/2, achieved through either gene-knockout or pharmacological treatment, leads to increased M1 polarization in a YAP-dependent manner, resulting in the development of M1-associated inflammatory disorders. Moreover, MST1/2 inhibition also leads to a substantial reduction in M2 polarization, but this occurs through the STAT6 and MEK/ERK signaling. The STAT6 is independent of YAP, but MEK/ERK is dependent of YAP. Consistent with these observations, both MST1/2-conditional knockout mice and wild-type mice treated with XMU-MP-1, a chemical inhibitor of MST1/2, exhibited reduced M2-related renal fibrosis, while simultaneously displaying enhanced LPS-mediated inflammation and improved clearance of MCR3-modified gram-negative bacteria. These findings uncover a novel role of MST1/2 in regulating macrophage polarization and establish it as a potential therapeutic target for the treatment of macrophage-related fibrotic diseases.

## Introduction

Macrophages are a critical component of host innate immunity that play an essential role in homeostasis, pathogen defense, and tissue repairmen 1. This multi-functionality often relies on the polarization that is further regulated by the cellular or tissue microenvironment. Specific cues from the tissue microenvironment can result in macrophage polarization toward a multitude of functional phenotypes 2. Traditionally, activated macrophages can be polarized into classically activated macrophages (M1) or alternatively activated macrophages (M2) 3. M1, which is activated by interferon-γ (IFN-γ) or lipopolysaccharides (LPS), produces pro-inflammatory cytokines such as interleukin-1 (IL-1) and tumor necrosis factor alpha (TNF-α), nitric oxide (NO), and reactive oxygen intermediates and promotes a polarized type I immune response to mediate host defense against bacterial, protozoa, and viral infection, as well as tumor cells 4-6. By contrast, M2, which responds to IL-4 and IL-13, displays anti-inflammatory function and promotes pathologic angiogenesis, organ fibrosis, tumor growth, or allergic and parasitic diseases 7. Thus, shifting the balance from M2 to M1 as therapeutic strategies may inhibit pathologic angiogenesis, fibrosis, or tumor growth 8-11. Nuclear factor kappa-light-chain-enhancer of activated B cells (NF-κB) and signal transducer and activator of transcription 6 (STAT6) are essential transcription factors in inflammatory and healing activation, respectively 12. The transcriptional response triggered by surrounding microenvironments, including cytokines, growth factors, and microorganism-associated molecular patterns, shapes the phenotype and function of macrophages 7. However, the intrinsic molecular mechanisms steering macrophage polarization have not been fully elucidated.

The mammalian sterile 20-like kinases 1 and 2 (MST1/2) are inhibitors of cell proliferation and promote apoptosis during development by inhibiting its downstream effectors YAP and TAZ through a kinase cascade formed by the scaffolding proteins WW45 and MOB1 and the kinases LATS1 and LATS2 13, 14. Growing evidence suggests that YAP is highly involved in inflammation-related diseases, such as inflammatory bowel disease and atherosclerosis 15, 16. The interaction between the NF-κB subunit p65 and YAP synergistically regulates the functions of immune cells 17. Importantly, the MST1/2 signaling pathway in mice participates in innate immune responses by CXCL1 and CXCL2 to regulate the production of antimicrobial peptides via the TLR-mediated pathway to fight *Mycobacterium tuberculosis* 18. MST1/2 acts in optimal ROS production and bactericidal activity of phagocytes by promoting the activation of the small GTPase Rac and mitochondrial trafficking and juxtaposition to the phagosome 19. However, limited studies have focused on the role of MST1/2 in macrophage polarization.

In the present study, the role of MST1/2 in M1 and M2 phenotypes of macrophages was investigated. MST1/2 is a regulator of M1 and M2 phenotypes of macrophages. MST1/2 knockout or inactivation increases M1-macrophage polarization and decreases M2-macrophage polarization. RNA-Seq analysis showed that MST1/2 inhibition induces distinct changes in cellular signaling in M1- and M2-type macrophages. The inhibition of MST1/2 promotes NF-κB p65 subunit nuclear accumulation by increasing the interaction of YAP and p65, and this process could induce M1-related inflammatory disorders, while the inhibition of MST1/2 decreased STAT6 (independent of YAP) and MEK/ERK pathway (dependent of YAP) to impair M2-type polarization. Mice with myeloid-specific deletion of MST1/2 exhibit enhanced M1 inflammatory response and spontaneously developed M1-related inflammatory disorders, but they are highly resistant to M2-related renal fibrosis. Overall, these findings define a key role for MST1/2 in limiting over-M1-related inflammation. The inactivation of MST1/2 could promote the clearance of the special LPS modified gram-negative bacteria (such as MCR-3 induced LPS modification decreases the sensitivity of macrophages to LPS) by increasing the inflammatory response of macrophage. The inactivation of MST1/2 could resist M2-related disease.

## Materials and methods

### Mice and ethics statement

*LysM^Cre^* (004781) and *Mst1/2^flox/flox^* mice (017635) mice with a C57BL/6J background were purchased from Jackson Laboratory. *LysM^Cre^* mice were crossed with *Mst1/2^flox/flox^* mice to generate *LysM^Cre^; Mst1/2^flox/-^* mice. Offspring mice were then hybridized with *Mst1/2^flox/flox^* mice to obtain *LysM^Cre^; Mst1/2^flox/flox^* after several generations of hybridization. Six- to eight-week-old *LysM^Cre^; Mst1/2^flox/flox^* (MST1/2 cKO) mice of female as experimental mice and *Mst1/2^flox/flox^* (MST1/2) mice as control mice. Six- to eight-week-old female WT C57BL/6J mice were purchased from Beijing Vital River Laboratory Animal Technology Co., Ltd for experiment.

All mice used for experiment were provided with a standard diet and were maintained under specific pathogen-free conditions barrier environment in the Laboratory Animal Center of China Agricultural University. All animal care and experimental protocols complied with the Animal Management Rule of the Ministry of Health, People's Republic of China and the Guide for the Care and Use of Laboratory Animals published by the United States National Institutes of Health. All subjections were approved by the Animal Care and Use Committee of China Agricultural University.

### Cell lines and bacterial strains

RAW 264.7 cell lines were cultured with high-glucose DMEM-supplemented media (Gibco) contained with 10% fetal bovine serum (Gibco), 1% penicillin streptomycin combination (Solarbio) and 1% L-glutamine (Hyclone), plated onto 10 cm cell culture dishes. RAW 264.7 cells were treated with 100ng/mL LPS (L2880, Sigma) or 10ng/mL IL-4 (214-14, PeproTech) and 10ng/mL IL-13 (210-13, PeproTech), and collected proteins for WB analysis.

*DH5α*-pHSG299 and *DH5α*-pHSG299-*mcr3* were constructed and stored in our laboratory. All *E. coli* strains were cultured with LB (LAND BRIDGE) containing Kanamycin.

### Isolation, culture and polarization of bone marrow derived macrophage (BMDM)

The femurs and tibias from mice were collected, and bone marrow cells were flushed with Hank's (Hyclone) contained with 5% penicillin streptomycin combination, then cell suspensions were filtered with 100μm cell sterile strainer (Falcon) for removing cell clumps. Cell precipitates were collected by centrifugation and suspended in Hank's. Cell suspensions treated with red blood cell lysis buffer (TIANGEN) for a few minutes and PBS (Hyclone) washed once. Finally, cell suspensions were suspended with RPMI 1640 Medium (Hyclone) contained with 20% fetal bovine serum, 1% penicillin streptomycin combination, 1% L-glutamine and 50ng/mL MCSF (315-02, PeproTech), plated onto 10 cm cell culture dishes. About after three days, cells were digested using trypsin, centrifuged to obtain cell precipitates. Cell precipitates were suspended with RPMI 1640 Medium contained with 10% fetal bovine serum, 1% penicillin streptomycin combination, 1% L-glutamine and 50ng/mL MCSF 19. After about two days BMDMs could be obtained. BMDMs were treated with 100ng/mL LPS (L2880, Sigma) + 20ng/mL IFN-γ (315-05, PeproTech) or 10ng/mL IL-4 (214-14, PeproTech) + 10ng/mL IL-13 (210-13, PeproTech), and collected mRNAs for qRT-PCR and RNA-Seq analysis. BMDMs were treated with 100ng/mL LPS or 10ng/mL IL-4 + 10ng/mL IL-13, and collected proteins for WB analysis. 20, 21

### Lung injury model by LPS

Mice were infected with 50mg kg^-1^ LPS or PBS (as solvent control), meanwhile infected with 2 mg kg^-1^ XMU-MP-1 (HY-100526, MedChemExpress) or DMSO (as solvent control) by intraperitoneal injection to establish the LPS-induced lung injury model and collected mice lungs after infection for 12h and 24h 22, 23. The lung tissues were fix in 4% paraformaldehyde (Solarbio) or placed in Trizol (Thermo Fisher). The lung tissues fixed in 4% paraformaldehyde were dehydrated using an alcohol gradient and then xylene was used to replace the alcohol. Lung tissues were embedded in paraffin by wax immersion. The lung tissues placed in Trizol were used for RNA extraction.

### Bacterial infection model

For survival assay, fresh cultures of *E. coli* were suspended in PBS. Mice were intraperitoneally (i.p.) injected with* mcr3*-positive/negative *E. coli* at doses of 1×10^9^ or 8×10^8^ CFU per mice at 0h. XMU-MP-1 group was intraperitoneally (i.p.) injected with 2 mg kg^-1^ XMU-MP-1 (dilution in DMSO) at 0 and 24h, Control group were intraperitoneally (i.p.) injected with same volume of DMSO solvent at 0 and 24h 24.

For bacterial clearance rate and cytokine level, fresh cultures of *E. coli* were suspended in PBS. Mice were intraperitoneally (i.p.) injected with* mcr3*-positive/negative *E. coli* at doses of 5×10^8^ CFU per mice at 0h. XMU-MP-1 group was intraperitoneally (i.p.) injected with 2 mg kg^-1^ XMU-MP-1 (dilution in DMSO) at 0 and 24h. Control group and MST1/2 cKO group were intraperitoneally (i.p.) injected with same volume of DMSO solvent at 0 and 24h. After 48h alive mice were chosen to dissection for collecting tissues placed in PBS or Trizol to determine bacteria load and inflammatory factors level 19.

### Unilateral Uretera Obstruction (UUO) model

Mice were subjected UUO surgery. Mice were general anesthetized with pentobarbital sodium (Sigma) through intraperitoneal injection and the left abdominal cavity was open for exposing left kidney and ureter. The ureter was ligated using 4-0 sterile surgical thread. Kidneys and ureters were put back into abdominal cavity. The wound was then sutured. Bilateral kidneys were collected on UUO 7 and 14 days 25. The kidneys were fixed in 4% paraformaldehyde (Solarbio) or placed in Trizol (Thermo Fisher). The kidney tissues fixed in 4% paraformaldehyde were dehydrated using an alcohol gradient and then xylene was used to replace the alcohol. The kidney tissues were embedded in paraffin by wax immersion. The kidney tissues placed in Trizol were used for RNA extraction 26**.**

### Inhibition

The kinase activity of MST1/2 was inhibited with XMU-MP-1 (HY-100526, MedChemExpress) as described in previous study 27. *In vitro*, BMDMs or RAW 264.7 cells were pretreatment with 3μM or 5μM XMU-MP-1 for 4-6h before other treatment. *In vivo*, 2 mg kg^-1^ XMU-MP-1 was administered to mice by intraperitoneal injection. For LPS-related mice experiments, as Figure 4A, XMU-MP-1 were injected into mice at 0h. For bacterial-related mice experiments, as Figure 5A, XMU-MP-1 were injected into mice at 0 and 24h. For UUO-related experiment, as Figure 8A, 2mg kg^-1^ XMU-MP-1 was administered or not to mice for six consecutive days after UUO. Verteporfin (VP, HY-B0146, MedChemExpress) was using as YAP inhibitor as described in previous study 28. *In vitro*, BMDMs were pretreatment with 1μM VP for 2-4h before other treatment.

### Histology and imaging

After anesthesia mice were infused with normal saline contained heparin sodium through the left ventricle, and lungs and kidneys were collected for paraffin sections according to the reagent instructions. The lung paraffin sections were stained with hematoxylin eosin stain (HE) (Solarbio) and immunohistochemistry (IHC). Kidney paraffin sections were stained with Sirius red stain (Solarbio) and immunohistochemistry. IHC was performed as described 29. In brief, sodium citrate antigen retrieval solution (Solarbio) was used for antigen retrieval of the sections. Then added serum-free protein block buffer (Solarbio) for 1h and 3% H_2_O_2_ (GeneTech (Shanghai) Company Limited) for 15min in dark. The sections incubated with anti-CD86 (1:200, 19589, Cell Signaling Technology) or anti-Arg-1 (1:200, 93668, Cell Signaling Technology) at 4 °C overnight. Subsequent antibody incubation and DAB color development were performed with GTVision^TM^+Detection System/Mo&Rb (GeneTech (Shanghai) Company Limited). These sections were visualized with a Nikon 80i microscope (Nikon). Collagen was visualized under polarized light. Images were analyzed by Nikon NIS-Elements Br 3.0 software (Nikon). To quantify the degrees of kidney fibrosis, we counted five fields for each mouse for analyzing in sections (n=3 mice per group). The value of fifteen fields were calculated as the per group data. The kidney section field we chose contained one or two glomeruli 24, 30.

### Cell immunofluorescence

BMDMs were placed in 6-well plates or cell slides. BMDMs were treatment with LPS with or without XMU-MP-1 and fixed with paraformaldehyde (Solarbio).

BMDMs in 6-well plates were incubated with anti-p65 antibody (1:500, 8242, Cell Signaling Technology) overnight at 4 °C. As secondary antibodies, Alexa 488 (Cell Signaling Technology) were added in well plates for 1h at room temperature, and then added Hoechst (TIANGEN) for 15 min in dark. These 6-well plates were visualized with a Nikon Ti2-E microscope (Nikon). Images were analyzed by Nikon NIS-Elements Br 3.0 software (Nikon). To quantify the nuclear translocation of p65, ten files from each group were analyzed for the p65 high positive in nuclei (%). The p65 high positive in the nuclei (%) was calculated (p65 high positive in the nuclei (%) = the number of p65 high positive in the nuclei ×100 / the number of total nuclei) 14.

BMDMs on cell slides were incubated with anti-p65(1:500, 8242, Cell Signaling Technology) and anti-YAP (1:50, sc-101199, Santa Cruz) antibodies. As secondary antibodies, Alexa 594 and 488 (Cell Signaling Technology) were added for 1h at room temperature in dark, and then sections were sealed by fluorescent mounting medium with DAPI (ZSGB-BIO) in dark. These sections were visualized with a Nikon 80i microscope (Nikon). Images were analyzed with Nikon NIS-Elements Br 3.0 software (Nikon).

### Real-time PCR analysis and RNA-Seq

RNA was extracted though the Trizol regent method (Thermo Fisher). 1000ng RNA was reverse transcribed to cDNA using HiScript III RT SuperMix (Vazyme). Aliquots of the reaction mixture was using for qRT-PCR analysis. The primers used in the experiments were *IL-1β* 5'-CAACCAACAAGTGATATTCTCCATG-3' 5'-GATCCACACTCTCCAGCTGCA-3', *IL-6* 5'-GGGAAATCGTGGAAATGAGA-3' 5'-TTCTGCAAGTGCATCATCGT-3', *TNF-a* 5'-TGAGCCCATATACCTGGGAG-3' 5'-CACCCATTCCCTTCACAGAG-3', *Arg-1* 5'-AACAC-GGCAGTGGCTTTAAC-3' 5'-GAGGAGAAGGCGTTT-GCTTA-3', *Fibronectin* 5'-TACCAAGGTCAATCCACACCCC-3' 5'-CAGATGGCAAAAGAAAGCAGAGG-3', *Collage-1* 5'-GCTGGTCTTCCAGGTCCTAAG-3' 5'-CGCCATCTTTGCCAGGAGAA-3', *Acta 2* 5'-GTCCCAGACATCAGGGAGTAA-3' 5'-TCGGATACTTCAGCGTCAGGA-3', *GAPDH* 5'-TGCCCCCATGTTTGTGATG-3' 5'-TGTGGTCATGAGCCCTTCC-3'. Relative gene expression was calculated from cycle threshold (Ct) values using GAPDH as an internal control (relative expression = 2^(sample Ct -GAPDH Ct)^). All data were normalized 31.

After XMU-MP-1 treatment for 6h, BMDMs treated with LPS + IFN-γ and IL-4 + IL-13 for 24h, and collected RNA to commissioned ANOROAD GENOME for transcriptome sequencing analysis. RNA-Seq data were subjected to KEGG analysis using Omicshare tools, volcano plots were performed using Omicstudio tools and heat maps were performed using GraphPad Prisim 8.

### Co-immunoprecipitation analysis

RAW264.7 cells were treated with LPS for 1h after adding XMU-MP-1. The cells were lysed using RIPA lysis buffer (Beyotime Biotechnology). Lysates were incubated with anti-p65 (1:100, 8242, Cell Signaling Technology) overnight at 4 °C. The sepharose beads (Santa Cruz) were added to the mixture, incubated for 2h at 4 °C. The beads were washed with ice PBS, and collected them for WB analysis.

### Immunoblotting analysis

Total proteins were extracted from cells and kidneys with RIPA lysis buffer (Solarbio). Nuclear and cytoplasmic proteins were extracted using the Nuclear and Cytoplasmic Protein Extraction kit (Beyotime Biotechnology). Proteins were separated by SDS-PAGE (GenStar), then transferred to NC membranes (MILLIPORE). NC membranes were incubated with the following primary antibodies (1:1000) overnight at 4 °C: anti-MST1 (ab51134, Abcam), anti-MST2 (ab236300, Abcam), anti-p-MOB1 (8699, Cell Signaling Technology), anti-p-YAP/TAZ (8418, Cell Signaling Technology), anti-p-p65 (3033, Cell Signaling Technology), anti-p65 (8242, Cell Signaling Technology), anti-p-p38 (9212, Cell Signaling Technology), anti-p-ERK (9101, Cell Signaling Technology), anti-Arg-1 (93668, Cell Signaling Technology), anti-p-STAT6 (ab263947, Abcam), anti-p-MEK (9154, Cell Signaling Technology), anti-Histone 3 (3638, Cell Signaling Technology), anti-GAPDH (TA-08, ZCGB BIO), anti-β-actin (TA-09, ZCGB BIO). DyLight 680 and 800-conjugated secondary antibodies (Cell Signaling Technology) incubated for 1h at room temperature. The members were analyzed by Azure Sapphire (Azure biosystems) 32.

### Measuring of bacterial loads in tissues

Hearts, livers, spleens, lungs, and kidneys were obtained aseptically from mice with *E. coli* infection. The tissues were weighed, placed in sterile PBS, and then homogenize. The homogenates were diluted in gradients and incubated on LB agar medium (LAND BRIDGE) at 37 °C for 18-24h. The total CFUs (colony-forming units) were counted in homogenates. CFU/g tissue = total CFUs / tissue weight (g) 5, 19.

### Schematics drawing

The schematics presented in this paper were provided by Adobe illustrator and Figdraw.

### Statistical analysis

GraphPad Prism 8 was used to conduct all statistical analyses. All data are expressed as means ± SEM. Specific sample sizes, statistical methods, and* P* values are indicated in Figures or Figure legends.

## Results

### MST1/2 is activated in polarizing macrophages

**Figure 1:**
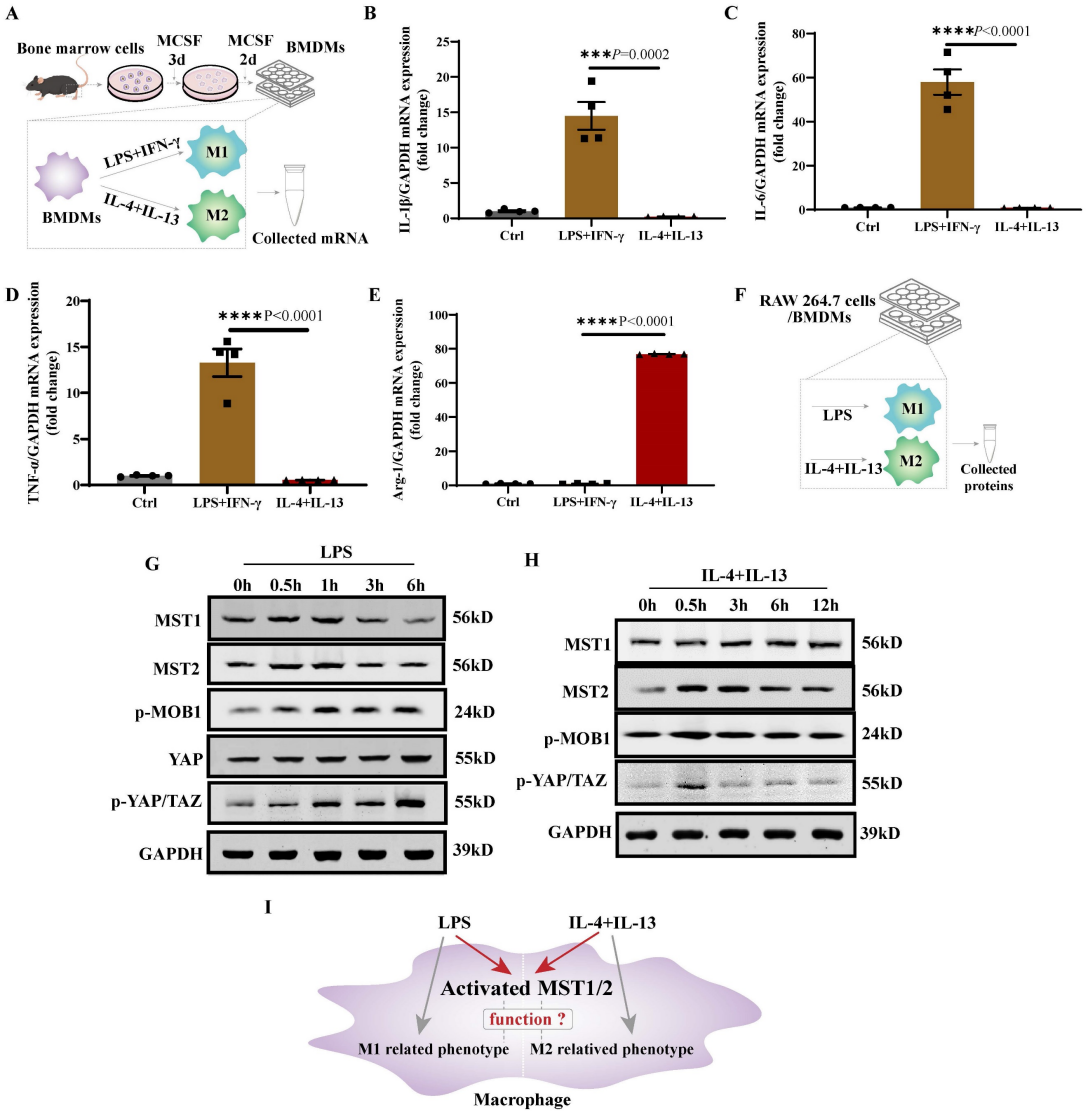
** MST1/2 was activated in polarizing M1 and M2-type macrophages.** (A) The protocol is BMDM production and polarization. Bone marrow cells were induced to BMDMs by MCSF. BMDMs were induced to M1 or M2-type macrophage by adding LPS (100ng/mL)+IFN-γ (20ng/mL) or IL-4 (10ng/mL)+IL-13 (10ng/mL). mRNA was collected for qRT-PCR. (B-E) The mRNA expressions of *IL-1β*, *IL-6*, *TNF-α* and *Arg-1* in BMDM were analyzed at 24 h post LPS+IFN-γ and IL-4+IL-13 treatment. GAPDH was used as the loading control. Data were presented as means ± SEM. Each symbol in the bar graphs indicates an individual data value. *P* values were calculated using *t* test comparing LPS+IFN-γ group to IL-4+IL-13 group, n=4 independent samples. (F)The RAW 264.7 cells/BMDMs were treated with LPS or IL-4+IL-13, at different time points the key proteins levels of Hippo pathway were determined by WB (G and H). GAPDH was used as the loading control. (I) LPS or IL-4+IL-13 treatment, the MST1/2 are activated in macrophage, but whether MST1/2 regulates macrophage polarization remains unclear.

LPS+IFN-γ promotes M1 polarization, while IL-4+IL-13 promotes M2 polarization 1. Bone marrow cells were collected, and then differentiated to mature bone marrow derived macrophages (BMDM) under treatment with MCSF 33. BMDMs were treated with LPS+IFN-γ or IL-4+IL-13, and their mRNA was collected for 24 h (Figure 1A). The mRNA expression levels of *IL-1β*, *IL-6*, *TNF-α*, and* Arg-1* in BMDM with LPS+IFN-γ or IL-4+IL-13 stimulation were determined using qRT-PCR. BMDM with LPS+IFN-γ stimulation expressed higher *IL-1β*,* IL-6*, and* TNF-α* and lower *Arg-1* levels than BMDM with IL-4+IL-13 stimulation (Figure 1B-E). The RAW 264.7 was similar result. Compared with untreated cells (Ctrl), RAW 264.7 with LPS or IL-4+IL-13 stimulation were higher mRNA level of polarized marker (Figure S1A-D). Then MST1 and MST2 protein levels and their activity in RAW 264.7/BMDM with LPS and IL-4+IL-13 stimulation were tested at different time points of treatments (Figure 1F). MST1 and MST2 in RAW 264.7 were elevated regardless of LPS and IL-4+IL-13 stimulation. MST1/2 activity is upregulated by phosphorylation 34. Subsequently, MOB1 is phosphorylated by them to elevate the phosphorylation of YAP and TAZ 35. Importantly, the p-MOB1 and p-YAP/TAZ of RAW 264.7 increased after treatment with LPS, and the parameters increased, and then decreased after IL-4+IL-13 treatment. (Figure 1G and H, Figure S1E-M). Similar results in BMDMs are shown in Figure 3B and 6B. It was previously reported that YAP protein level was increased after LPS inducing to regulate M1 macrophage 17. We checked YAP protein level after LPS treatment. Similar as MST1 and MST2, YAP was increased (Figure 1 G). These results indicate that MST1/2 might contribute to the regulation of macrophage polarization (Figure 1I), but their functions are unclear.

### Blocking MST1/2 activation enhances M1 polarization and reduces M2 polarization

**Figure 2:**
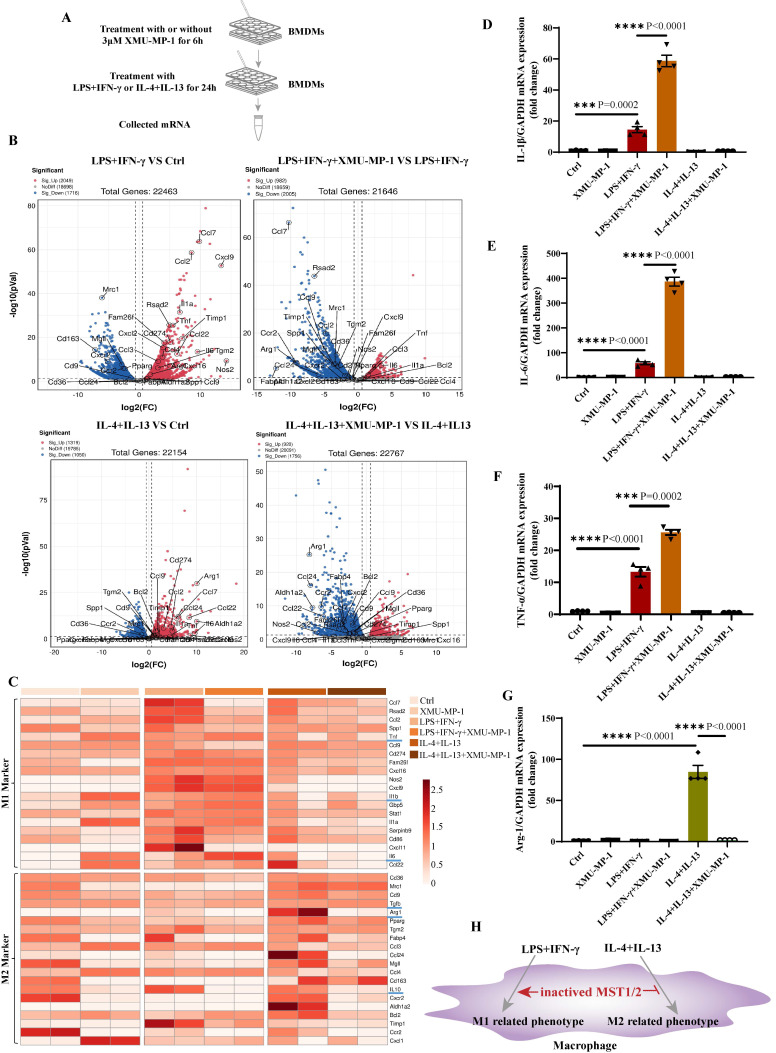
** MST1/2 inhibition promoted M1-type polarization but impaired M2-type polarization.** (A) BMDMs were treated with or without 3μM XMU-MP-1 for 6h, then adding LPS (100ng/mL)+IFN-γ (20ng/mL) or IL-4 (10ng/mL)+IL-13 (10ng/mL) for 24h. mRNA was collected for RNA-Seq and qRT-PCR. (B) Volcano plot of the RNA-Seq data of LPS+IFN-γ treatment versus control group, LPS+IFN-γ with XMU-MP-1 versus LPS+IFN-γ, IL-4+IL-13 versus control group or IL-4+IL-13 with XMU-MP-1 versus IL-4+IL-13, n=2 independent samples. (C) Heap map showed the expressions of M1- and M2- markers of RNA-Seq data, n=2 independent samples. (D-G) The mRNA expressions of *IL-1β*, *IL-6*, *TNF-α* and* Arg-1* were analyzed by qRT-PCR with different treatment. GAPDH was used as the loading control. Data were presented as means ± SEM. Each symbol in the bar graphs indicates an individual data value. *P* values were calculated using *t* test compared with control group or between the indicated groups, n=4 independent samples. (H) In WT macrophage, MST1/2 is activated under LPS or IL-4+IL-13 treatment. But when MST1/2 activation is inhibited, M1 related-inflammatory phenotypes are increased and M2 related-phenotypes were decreased.

To address the role of MST1/2 in macrophage polarization, we used XMU-MP-1, a specific inhibitor of MST1/2, to block MST1/2 activation 27. Studies have shown that XMU-MP-1 could significantly inhibit the activation of MST1/2 and its downstream molecules (MOB1, LATS, and YAP) 27, 36-38. BMDM is a well-established system that can be used to assess macrophage polarization the first 24 h of treatment with LPS+IFN-γ or IL-4+IL-13 is the phase of polarization induction 39. Accordingly, XMU-MP-1 was first added for 6 h, followed by 24 h of treatment with LPS+IFN-γ or IL-4+IL-13. Then, mRNA was collected for RNA-Seq and qRT-PCR (Figure 2A). Genes upregulated or downregulated with or without XMU-MP-1 under LPS+IFN-γ or IL-4+IL-13 (fold change > 1.5) were shown. The volcano map is shown in Figure 2B, and the M1 and M2 markers were used to generate the heat map shown in Figure 2 C. In comparison with main M1 and M2 markers in BMDM with or without XMU-MP-1, the gene expression of macrophages only treated with XMU-MP-1 were not found universal change towards M1 or M2. In comparison with the control group, M1 markers were increased universally in macrophages with LPS+IFN-γ with or without XMU-MP-1 addition. In comparison with the control group, M2 markers were increased universally in macrophages only with IL-4+IL-13, but the addition of XMU-MP-1 in macrophages with IL-4+IL-13 M2 markers were reduced universally compared with macrophages with IL-4+IL-13. When inhibiting MST1/2 in macrophages with LPS+IFN-γ, these M1 markers (TNF-α, IL-1, and IL-6), which are important to pro-inflammation, were increased, whereas some M1 markers were decreased (Figure 2B and C). To confirm these findings, we used qRT-PCR to test the mRNA levels of *IL-1β*, *IL-6*, *TNF-α* and *Arg-1* in macrophages with above each treatment, and the results are similar to the RNA-Seq data (Figure 2D-G). Therefore, inhibiting MST1/2 by XMU-MP-1 promote inflammatory M1 polarization but impair M2 polarization (Figure 2H). MST1/2 could limit over inflammatory response to LPS/IFN-γ, but promote anti-inflammatory response to IL-4/IL-13.

### RNA-Seq analysis for potential regulatory pathway of MST1/2 inhibition in macrophage polarization

Gene set enrichment analysis and functional pathway of mRNA datasets show the pathways with distinctly activated or downregulated M1- and M2-type polarization. Among the 30 most significant upregulated KEGG pathways that mainly involved the enrichment of M1-type polarization are TNF signaling, NOD-like receptor signaling, cytokine-cytokine receptor interaction, HIF-1 signaling, chemokine signaling, JAK-STAT signaling, TLR signaling pathway and down-regulated pathway are FoxO signaling, MAPK signaling, metabolic pathway (Figure S2A). In comparison with M1-type polarization with XMU-MP-1 to M1-type polarization without XMU-MP-1, among the 30 most significant up-regulated KEGG pathways that were predominantly enrichment were Toll-like receptor signaling, TNF signaling and metabolic pathway, while the downregulated KEGG pathways were cytokine-cytokine receptor interaction, NOD-like receptor signaling, TNF signaling, chemokine signaling, Toll-like receptor signaling, PPAR signaling and metabolic pathways (Figure S2B). Among the 30 most significant upregulated KEGG pathways that were predominantly enriched, among the M2-type polarization were JAK-STAT signaling, HIF-1 signaling, cytokine-cytokine receptor interaction, oxidative phosphorylation, TNF signaling pathway, chemokine signaling and metabolic pathways. The down-regulated KEGG pathways were MAPK signaling, FoxO signaling, Hippo signaling pathway-multiple species, Notch signaling, chemokine signaling, PI3K-Akt signaling, cytokine-cytokine receptor interaction and metabolic pathways (Figure S2C). In comparison with M2-type polarization with XMU-MP-1 to M2-type polarization without XMU-MP-1, among the 30 most significant up-regulated KEGG pathways that were predominantly enrichment were HIF-1 signaling, AMPK signaling, p53 signaling, Notch signaling, MAPK signaling pathways, metabolic pathways, and the downregulated KEGG pathways were cytokine-cytokine receptor interaction, chemokine signaling, HIF-1 signaling and metabolic pathways (Figure S2D). Collectively, these RNA-Seq data provide a comprehensive resource to link similar inflammation related signaling pathway by XMU-MP-1 treatment in M1- and M2-type macrophage polarization.

### Deletion of MST1/2 increases p65 accumulation in nucleus under LPS stimulation

**Figure 3:**
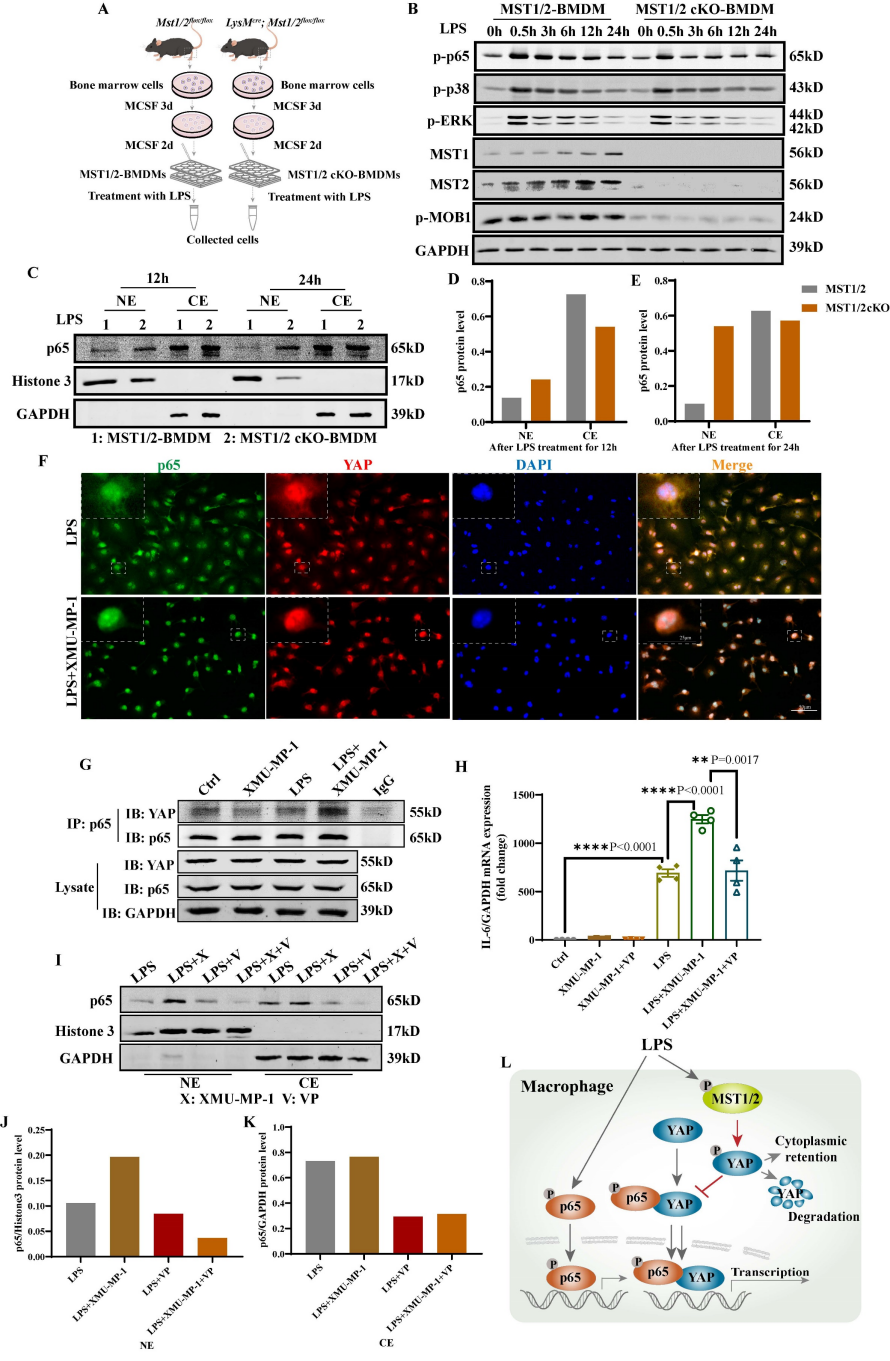
** Deficient MST1/2 did not affect p65, p38-MAPK and ERK activated, but increased p65 accumulation in nucleus by elevating interaction of YAP and p65.** (A) MST1/2-BMDMs and MST1/2 cKO-BMDMs from *Mst1/2^flox/flox^* (MST1/2) mice and *LysM^Cre^; Mst1/2^flox/flox^* (MST1/2 cKO) mice were collected, treated for different time by LPS (100ng/mL) and collected cells. Cells were prepared for WB and IF. (B) The p-p65, p-p38, p-ERK, MST1, MST2 and p-MOB1 protein levels of MST1/2- and MST1/2 cKO-BMDMs were analyzed by WB after LPS treatment for 0, 0.5, 3, 6, 12 and 24h. GAPDH was used as the loading control. (C) The p65 protein levels of MST1/2- or MST1/2 cKO-BMDMs were analyzed in nucleus and cytoplasm by WB after LPS induction for 12h and 24h. The p65 protein levels in the nucleus and cytoplasm (D and E) were calculated. Histone 3 and GAPDH were used as the nuclear and cytoplasmic loading control. (F) The immunofluorescent staining of p65 and YAP in BMDMs with or without XMU-MP-1 were conducted after LPS treatment 12h. Scale bars = 50 μm. (G) The co-immunoprecipitation analysis of p65 and YAP in RAW264.7 cells were treated with LPS for 1h after adding XMU-MP-1. Lysate was immunoprecipitated with anti-p65. Immunocomplex was subjected to western blotting using anti-YAP, anti-p65, lysate was using anti-YAP, anti-p65 and anti-GAPDH. (H) The *IL-6* mRNA level was analyzed by qRT-PCR after XMU-MP-1 and/or VP pre-treatment and IL-4+IL-13 treatment for 24h. GAPDH was used as the loading control, n=4 independent samples. (I) The p65 protein levels were analyzed in nucleus and cytoplasm by WB after LPS induction for 24h with or without XMU-MP-1 and/or VP. The p65 protein levels in the nucleus and cytoplasm (J and K) were calculated. Histone 3 and GAPDH were used as the nuclear and cytoplasmic loading control. (L) In macrophage, p65/YAP complex enhances p65 entering nucleus to drive inflammatory respond after LPS treatment. Meanwhile MST1/2 is activated, and activated MST1/2 decreases p65/YAP complex accumulation in the nucleus by phosphorylating YAP to limit inflammatory level.

To investigate the role of MST1/2 in macrophage polarization *in vitro* and *in vivo*, we generated mice with MST1/2 deletion, specifically in myeloid cell by crossing *Mst1/2^flox/flox^* mice with transgenic mice that carry lysozyme (*LysM*) proximal promoter-mediated Cre recombinase, in which the target gene was specifically ablated from macrophages and neutrophils 40. The mice with MST1/2 homozygous deletion in myeloid cells (*LysM^Cre^; Mst1/2^flox/flox^*) were established by several rounds of crossing (Figure S3A) 40, 41. We collected bone marrow cells of *Mst1/2^flox/flox^* (MST1/2) or *LysM^cre^; Mst1/2^flox/flox^* (MST1/2 cKO) mice, and then induced to BMDMs by using MCSF. BMDMs were treated with LPS for different times, and cell protein was collected (Figure 3A). Figure 3B shows that in BMDM from the cKO mice, the MST1 and MST2 protein disappeared, and p-MOB1 decreased. LPS activates several signaling pathway, causing inflammatory factors in macrophages, such as NF-κB, p38 and ERK MAPK 42. Our RNA-Seq results have shown that inhibiting MST1/2 could affect these pathways (Figure S2B). These target proteins were detected using Western blot assays. LPS induced NF-κB, p38 and ERK MAPK activation by phosphorylation in both MST1/2 and MST1/2 cKO BMDMs, and almost same levels of protein activation was observed between MST1/2 and MST1/2 cKO (Figure 3B and S3B-D). Meanwhile, inhibition of MST1/2 did not affect the total protein levels of p65, p38, ERK and MOB1 (Figure S3E). NF-κB accumulation in nucleus increase transcription signal and interaction between the NF-κB subunit p65 and YAP synergistically regulate the functions of immune cells 19. To test whether MST1/2 cKO affect NF-κB subunit p65 accumulation in nucleus under LPS stimulation, we detected the p65 protein in the nucleus of BMDMs by using WB and IF. The p65 protein in the nucleus of MST1/2 cKO BMDMs was elevated at 12 h and 24 h post-LPS stimulation compared with MST1/2 BMDMs (Figure 3C-E), while the total p65 protein levels were not obvious difference in WT- and MST1/2 cKO- BMDM (Figure S3F). To confirm this finding, we used the MST1/2 inhibitor, and the BMDMs were immunostained with the p65 antibody at 0, 12, and 24 h post-LPS stimulation; then, the percentage of cells with p65 high positive in nuclei were counting (Figure S4A). In comparison with the control group, XMU-MP-1 did not affect the percentage of p65 positive in BMDMs nucleus without LPS treatment. At 12 and 24 h post-LPS stimulation, MST1/2 inhibition remarkably increased the percentage of p65 high positive in BMDM nucleus (Figure S4B and C). Therefore, MST1/2 did not affect LPS-induced NF-κB, p38 and ERK MAPK phosphorylation, but MST1/2 inhibition could increase p65 accumulation in nucleus. Yang et al. already demonstrated that LPS treatment induce an interaction of NF-κB p65 and YAP to and nuclear translocation in macrophages 17. To test YAP interaction with p65, we performed immunofluorescence and Co-IP. The IF result showed that inhibiting MST1/2 could collect p65 and YAP in the nucleus (Figure 3F). The Co-IP result further showed MST1/2 inhibition increased YAP and p65 interaction under LPS treatment (Figure 3G). To further test whether MST1/2 affects M1 polarization through YAP, we used a YAP inhibitor (VP) to inhibit YAP to detect macrophage polarization and nuclear accumulation of p65. The results showed compared with LPS+XMU-MP-1, the M1 marker (IL-6) mRNA significantly decreased after adding XMU-MP-1 and VP (Figure 3H). In both LPS+VP and LPS+XMU-MP-1+VP, the p65 protein levels in the nuclear and cytoplasm were lower than that in group LPS+XMU-MP-1 (Figure 3 I-K). The results indicated inhibition YAP could decrease p65 protein level in the nuclear and cytoplasm with or without inhibition of MST1/2. Thus, MST1/2 deletion did not directly activate LPS downstream signaling, but they could increase NF-κB signaling accumulation in the nucleus by elevating YAP interaction with p65 (Figure 3L).

### Inhibition of MST1/2 increased the pro-inflammatory factors in LPS-induced lung injury

**Figure 4:**
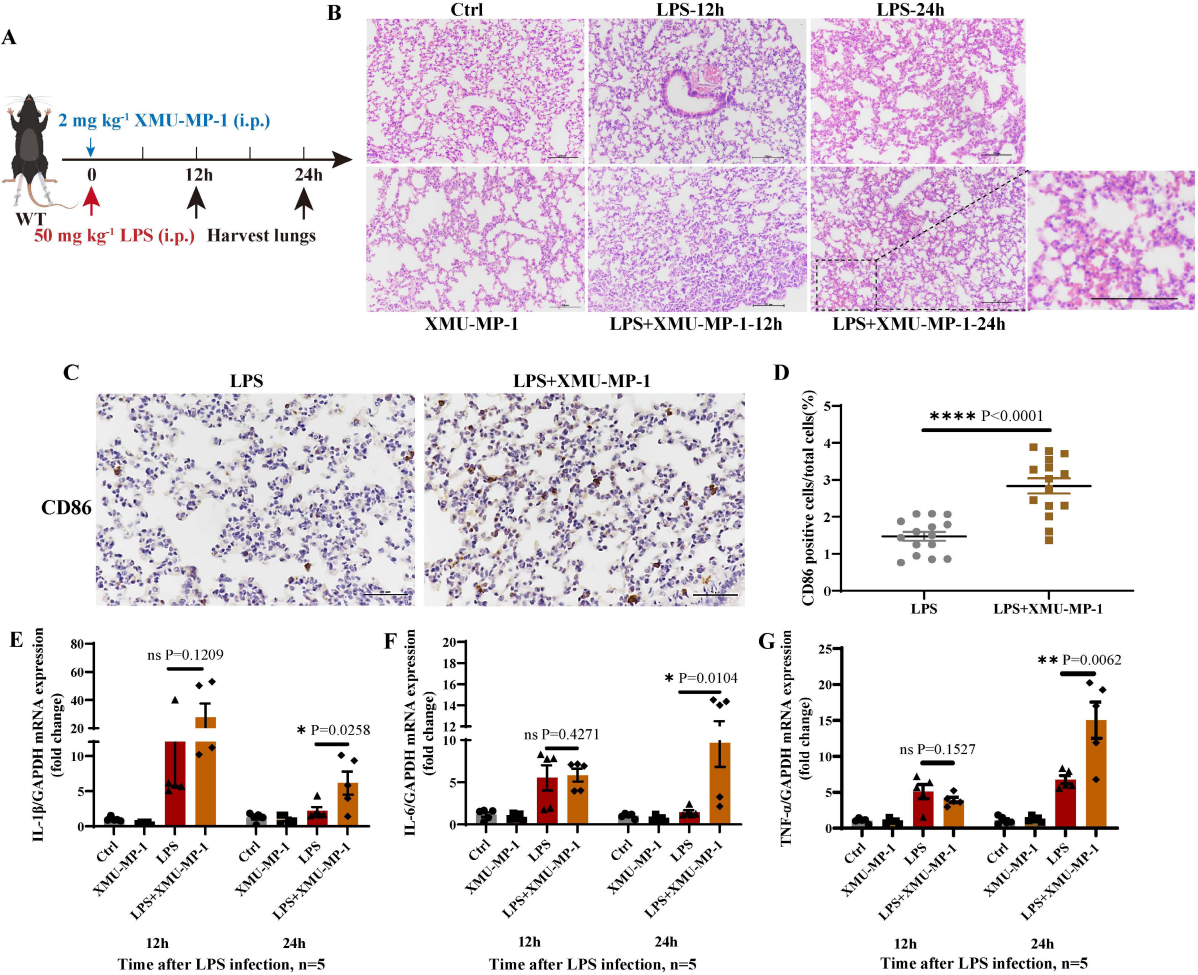
** Deficient of MST1/2 increased inflammatory respond in LPS induced lung injury** (A) The protocol of LPS infection experiment. (B) HE analyzed lungs injury of mice after LPS infection 12 and 24 h. Scale bars = 100 μm. (C) Immunohistochemistry analyzed the percent of CD86 positive cells in lungs of mice after LPS infection 12h. Scale bars = 50 μm. (D) CD86 positive cells/total cells was count. Each group consisted of three mice and five fields per mouse, n=15 fields per group. (E-G) The *IL-1β*,* IL-6* and *TNF-α* mRNA levels were determined by qRT-PCR after LPS infection 12 and 24 h. GAPDH was used as the loading control, n=5 mice per group. Data are presented as means ± SEM. Each symbol in the bar graphs indicates an individual data value. *P* values were calculated using *t* test.

To further determine the effect of MST1/2 inhibition on inflammatory microenvironment *in vivo* in mice, we used an acute endotoxin shock model (Figure 4A). Pathological test at 12 and 24 h showed that the lung of LPS+XMU-MP-1-treated mice had more severe inflammatory response and tissue damage than the mice treated with LPS only (Figure 4B). To check macrophage polarization in lung, we performed immunohistochemistry with anti-CD86. The IHC result showed that the percent of CD86 positive cells in lungs of LPS+XMU-MP-1-treated mice was increased (Figure 4 C and D). Meanwhile, the mRNA expression levels of *IL-1β*, *IL-6*, and *TNF-α* were tested in the lung of mice at 12 and 24 h post-LPS treatment. XMU-MP-1 treatment partly increased the LPS-induced *IL-1β*, *IL-6*, and *TNF-α* mRNA expression of lung at 24 h post-LPS treatment (Figure 4E-G). The inhibition of MST1/2 could enhance LPS-induced inflammation and promote the polarization of macrophages to M1 macrophage, but it may not directly activate LPS downstream pathway.

### Inhibition of MST1/2 increases the clearance rate of the *mcr3-*positive bacteria *in vivo* by elevating inflammatory factor release

**Figure 5:**
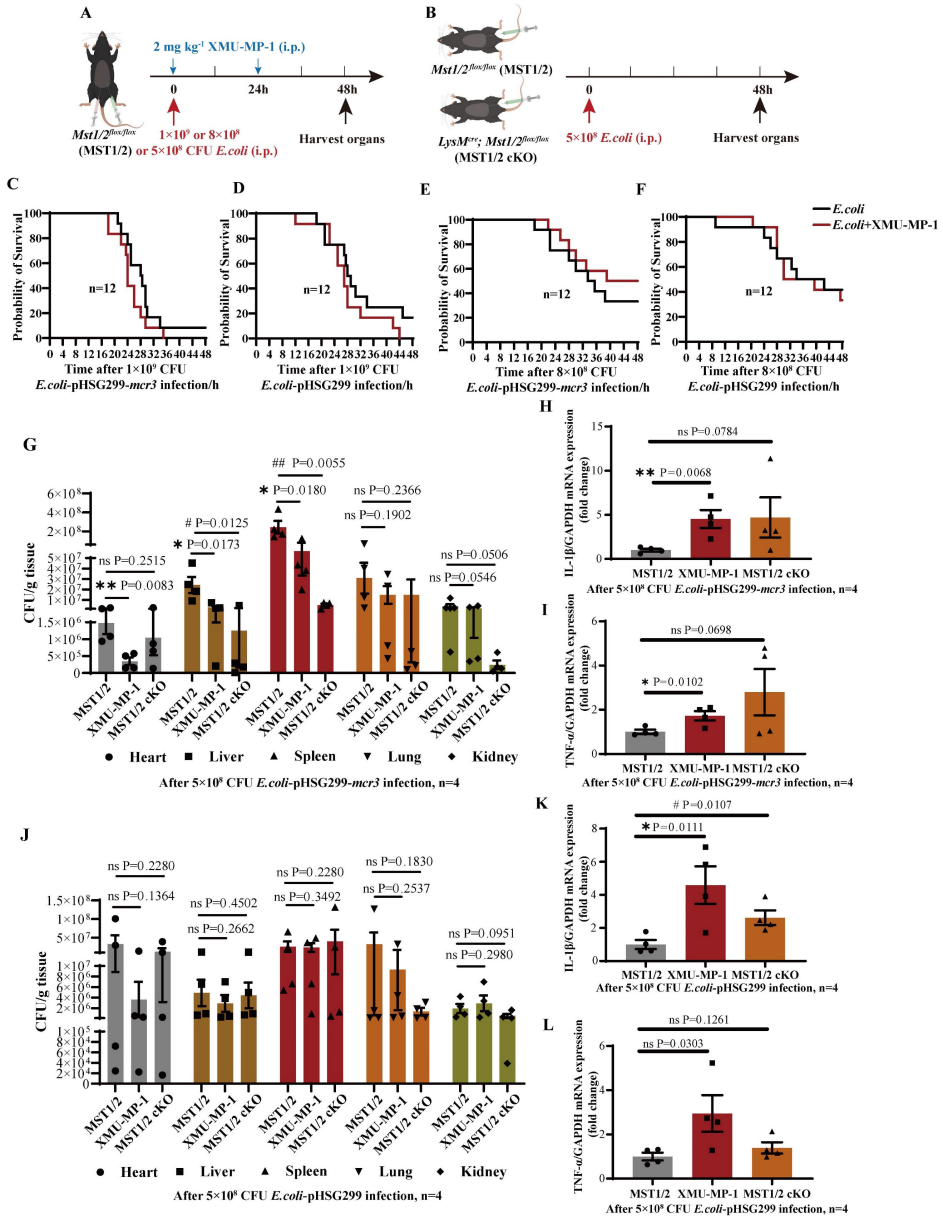
** The clearance rate of the *mcr3*-positive bacteria *in vivo* by elevating inflammatory factors release.** (A and B) The protocol of *E.coli* infection experiment. (C-F) The survival of mice with 1×10^9^ and 8×10^8^ CFU *E.coli* infection were showed, n=12 mice per group. (G and J) The bacteria load (as CFU/g tissue) in heart, liver, spleen, lung and kidney of mice with 5×10^8^ CFU* E.coli* infection for 48h were determined. (H, I, K, L) The *IL-1β* and *TNF-α* mRNA levels were determined by qRT-PCR after 5×10^8^ CFU *E.coli* infection for 48h. GAPDH was used as the loading control. Data were presented as means ± SEM. Each symbol in the bar graphs indicates an individual data value. *P* values were calculated using *t* test, n=4 mice per group. * compared XMU-MP-1 and MST1/2 group, # compared MST1/2 cKO and MST1/2 group.

Mobile colistin resistance enzyme (MCR-3) is a phosphoethanolamine transferase that modifies lipid A in gram-negative bacteria 43. *mcr3*-positive bacteria could increase their mortality in mice by reducing LPS-induced sensitivity of macrophages via LPS modified by phosphoethanolamine transferase 24. The effect of MST1/2 inhibition on the survival rate of the *mcr3*-positive/negative bacteria (*E.coli*-pHSG299-*mcr3/E.coli*-pHSG299) infected mice was tested using the XMU-MP-1-treated mice challenged with *mcr3*-positive/negative *E. coli*. Mice were challenged with 200 μL of *mcr3*-positive/negative *E. coli* at two different concentrations (1×10^9^ and 8×10^8^ colony-forming units (CFUs) per mice) with or without XMU-MP-1 (Figure 5A). At a concentration of 1×10^9^, more than 90% mice died in 48 h regardless of challenge with *mcr3*- positive/negative *E. coli,* and XMU-MP-1 treatment increased the mortality rates in mice (100%; Figure 5C and D). Therefore, a high concentration of bacteria stimulated a strong inflammatory response, causing severe sepsis and death, and XMU-MP-1 treatment enhanced inflammatory response to promote this process. This finding also supports that MST1/2 inhibition increases LPS-induced lung injury by enhancing inflammation. At a low dose of *E. coli* (8×10^8^ CFUs per mice), XMU-MP-1 treatment increased the survival rate of the mice infected with *mcr3*-positive bacteria, while XMU-MP-1 treatment did not affect the survival rate of the mice infected with *mcr3*-negtive bacteria (Figure 5E and F). This result supports that properly increased inflammatory reaction could reduce mouse death caused by *mcr3*-positive bacteria. To confirm this hypothesis, XMU-MP-1-treated mice and MST1/2 cKO mice challenged with *mcr3*-positive/negative *E. coli* with a lower dose (5×10^8^ CFUs per mice) was used to test the bacterial load of tissues and inflammatory factors at 48 h post-infection (Figure 5B). XMU-MP-1 treatment and MST1/2 cKO could decrease *mcr3*- positive bacteria load of tissues, especially in the liver and spleen, and a high mRNA expression of inflammatory factors was exhibited in the liver (Figure 5G-I). However, XMU-MP-1 treatment and MST1/2 cKO did not affect the *mcr3*-negative bacteria load of tissues, and inflammatory factors were highly expressed in the liver (Figure 5J-L). These results show that the inhibition of MST1/2 could enhance bacteria-induced inflammation. Some bacteria reducing LPS-induced sensitivity of macrophages, such as* mcr3*- positive bacteria, were partly impaired.

### MST1/2 controls M2 polarization by effecting the phosphorylation of STAT6 and MEK/ERK independent of YAP and dependent of YAP, respectively

**Figure 6:**
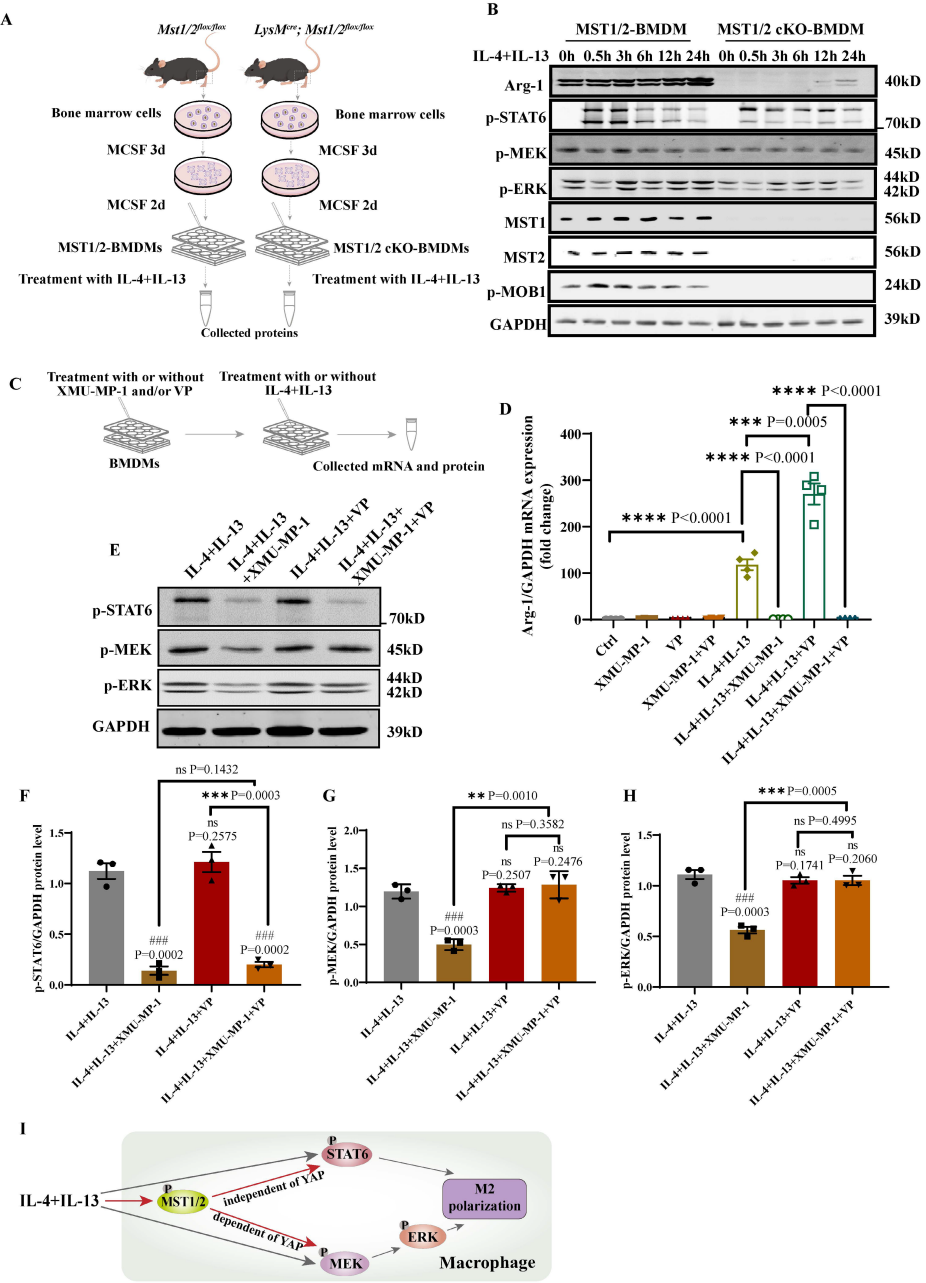
** Deficiency of MST1/2 decreased M2 polarization by down-regulating phosphorylation STAT6 and MEK/ERK independently of YAP.** (A) MST1/2 and MST1/2 cKO-BMDMs were collected, treated for different time by IL-4 (10ng/mL)+IL-13 (10ng/mL) and total cell proteins were collected for WB. (B) The Arg-1, p-STAT6, p-MEK, p-ERK, MST1, MST2 and p-MOB1 proteins levels of MST1/2- or MST1/2 cKO-BMDMs were analyzed by WB after IL-4+IL-13 treatment for 0, 0.5, 3, 6, 12 and 24h. GAPDH was used as the loading control. (C) The BMDMs treated with or without XMU-MP-1 or/and VP before IL-4+IL-13 treatment, collected mRNA and protein for qRT-PCR and WB. (D) The *Arg-1* mRNA level was analyzed by qRT-PCR after IL-4+IL-13 treatment for 24h. GAPDH was used as the loading control, n=4 independent samples. (E-H) The p-STAT6, p-MEK and p-ERK protein levels were analyzed by WB and the bands were counted after IL-4+IL-13 treatment for 3h. * compared two different groups as indicated. # compared IL-4+IL-13 group. GAPDH was used as the loading control. Data were presented as means ± SEM. Each symbol in the bar graphs indicates an individual data value. *P* values were calculated using *t* test. (I) In macrophage, IL-4+IL-13-activated MST1/2 up-regulates phosphorylation STAT6 independent of YAP, but up-regulates MEK/ERK via YAP.

The role of MST1/2 in IL-4/IL-13-induced M2 polarization has not been reported. Our RNA-Seq data and qRT-PCR results showed that the inhibition of MST1/2 reduced the mRNA expression of hallmarks of M2 macrophages (Figure 2B, C, and G). STAT6 and MEK/ERK pathway are the key signaling pathways of M2 polarization under IL-4/IL-13 stimulation 21. Arg-1 (a M2 hallmark), p-STAT6, p-MEK, and p-ERK protein levels were determined in BMDMs isolated from MST1/2 and MST1/2 cKO mice at 0, 0.5, 3, 6, 12, and 24 h post IL-4/IL-13 treatment (Figure 6A), and MST1/2 cKO significantly decreased the level of p-STAT6, p-MEK, p-ERK, and Arg-1 (Figure 6B and S5A-D). The previous report showed that YAP could promote macrophage polarization, but did not affect p-STAT6 15. To explore whether MST1/2 regulated M2 polarization through YAP, we used XMU-MP-1 and verteporfin (VP, YAP inhibitor) before IL-4+IL-13 treatment.

Then collected mRNA and protein to determined M2 marker (Arg-1) mRNA level and p-STAT6, p-MEK, p-ERK protein levels by qRT-PCR and WB (Figure 6C). The qRT-PCR showed that, compared with IL-4+IL-13 group, the *Arg-1* mRNA level of IL-4+IL-13+XMU-MP-1 group was significantly decreased and IL-4+IL-13+VP group was increased (Figure 6D). It was same as Figure 2C, G and the report 15. But compared IL-4+IL-13+VP group, after adding XMU-MP-1, the mRNA expression of *Arg-1* was significantly decreased. The protein levels of p-STAT6, p-MEK and p-ERK in macrophage was significantly decreased after adding XMU-MP-1 in IL-4+IL-13. And these protein levels were not change after adding VP (Figure 6E-H). Compared with IL-4+IL-13+XMU-MP-1 group, p-STAT6 was not change in the group of adding XMU-MP-1 and VP (Figure 6E and F), but p-MEK and p-ERK protein levels were significantly increased in the group of adding XMU-MP-1 and VP (Figure 6E, G and H). The levels of p-MEK and p-ERK were similar to Ctrl (Figure 6E, G and H). These results indicated that IL-4+IL-13-activated MST1/2 up-regulates phosphorylation STAT6 independent of YAP, but up-regulates MEK/ERK via YAP (Figure 6I).

### MST1/2 inhibition reduces UUO-induced renal fibrosis

**Figure 7:**
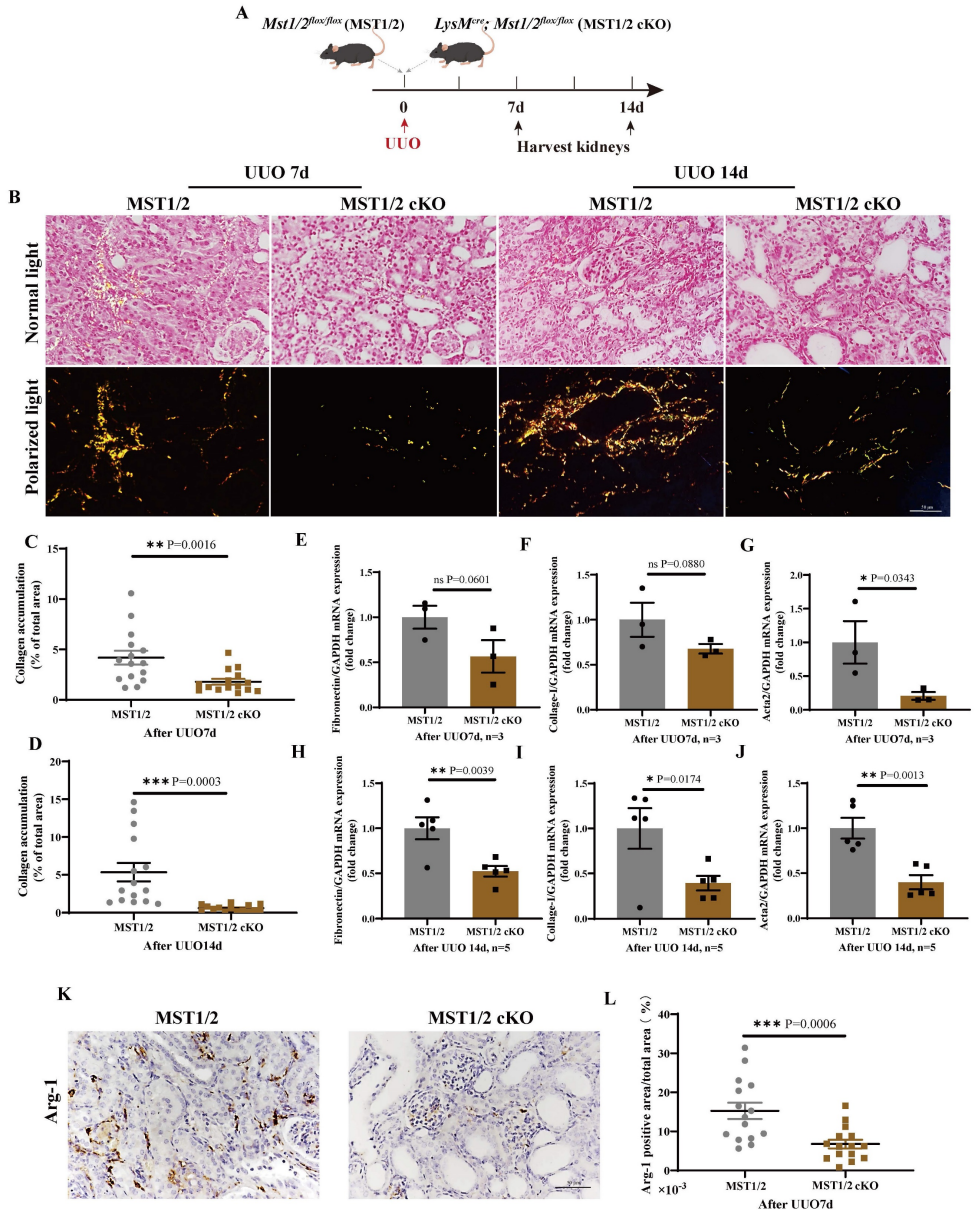
** MST1/2 knockout alleviated renal fibrosis**. (A) MST1/2 and MST1/2 cKO mice were performed UUO. At UUO 7d and UUO 14d, collected bilateral kidneys. (B) Obstructed kidney cross-sections were Sirius red staining after UUO 7d and 14d. Scale bars = 50 μm. (C and D) The percent of collagen accumulation area was calculated after UUO 7d and 14d. (E-J) The mRNA expressions of *Fibronectin*, *Collage-I*, and *Acta 2* in obstructed kidney were analyzed after UUO 7d (E-G, n=3 mice per group) and UUO14d (H-J, n=5 mice per group). GAPDH was used as the loading control. (K and L) Immunohistochemistry of protein expression of Arg-1 and bar graph showed quantification of areas of positive cells. Data were presented as means ± SEM. Each symbol in the bar graphs indicates an individual data value. *P* values were calculated using *t* test. * compared MST1/2 cKO and MST1/2 group.

Renal fibrosis is strongly related to M2 polarization. The effect of MST1/2 deletion in macrophage on M2 polarization and renal fibrosis *in vivo* was determined by performing a standard renal fibrosis model of mice (unilateral uretera obstruction, UUO model) 41. cKO mice were used to verify the role of macrophage-MST1/2 in renal fibrosis. Figure 7A shows the results of UUO on MST1/2 and MST1/2 cKO mice; kidneys were collected at 7 and 14 days after surgery. Sirius red staining demonstrated accumulation of collagen, which decreased in the injured kidneys of MST1/2 cKO mice at UUO 7 and 14 days (Figure 7B-D). Based on Sirius red staining, the mRNA expression of fibrotic markers (*Fibronectin, Collage-I, Acta2*) decreased in the injured kidneys of MST1/2 cKO mice compared with MST1/2 mice at either UUO 7 or 14 day (Figure 7E-J). In order to examine macrophage polarization, we performed immunohistochemistry of Arg-1 (M2 marker). The IHC showed the Arg-1 positive area was decreased in UUO kidneys of MST1/2 cKO mice compared MST1/2 mice (Figure 7K and L).

**Figure 8:**
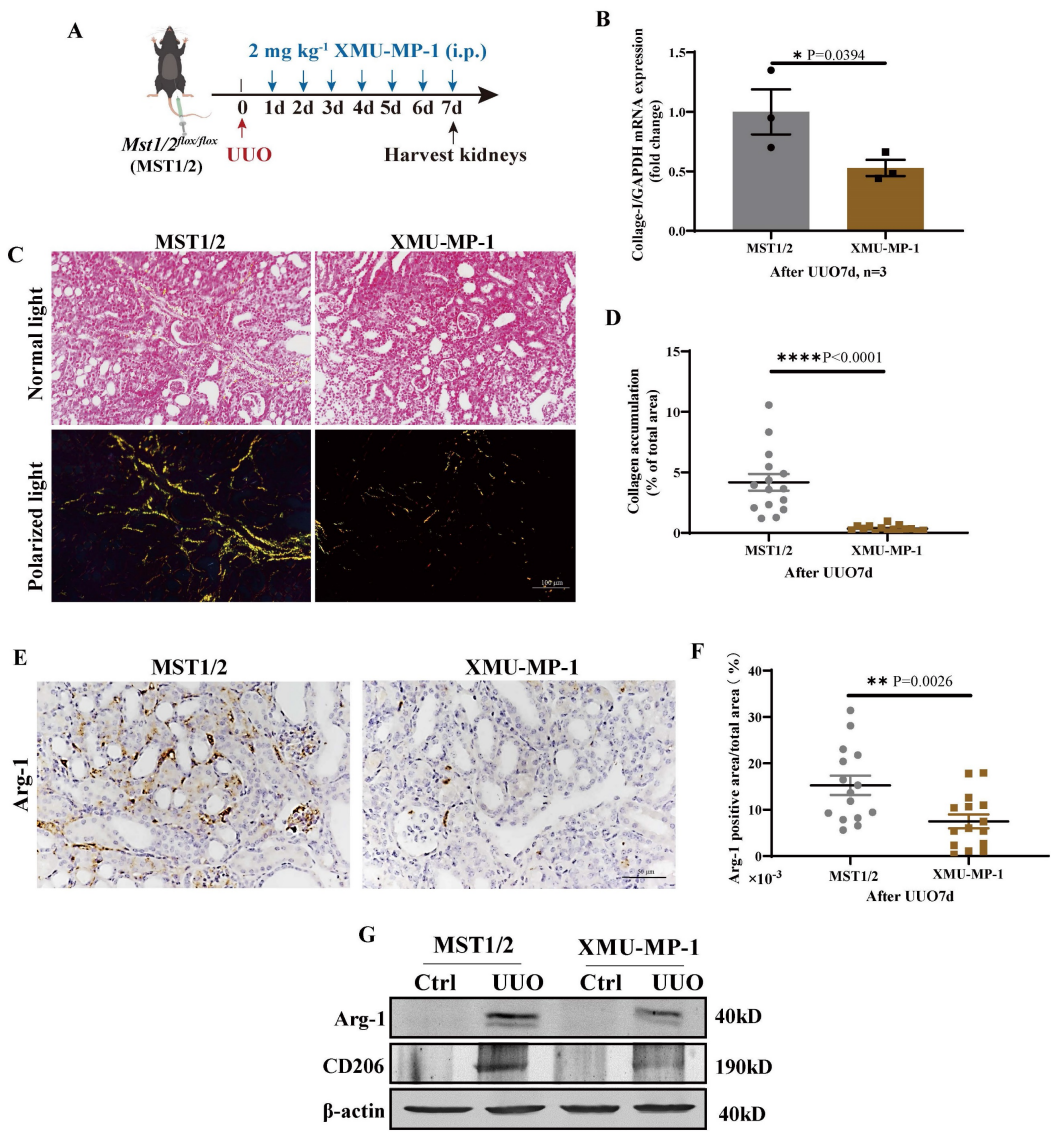
** Inhibition of MST1/2 impaired UUO-induced renal fibrosis.** (A) WT (MST1/2) mice were performed UUO at 0 day. Thereafter, 2mg kg^-1^ XMU-MP-1 was administered intraperitoneally for six consecutive days. After UUO 7 day, kidneys were collected. (B) The* Collage-I* mRNA expression level in obstructed kidney was determined on day 7 after UUO with or without XMU-MP-1 by qRT-PCR (n=3 mice per group). (C and D) Obstructed kidney cross-sections were performed Sirius red staining, and the percent of collagen accumulation area was calculated. Scale bars = 100 μm. (E and F) Immunohistochemistry of protein expression of Arg-1 and bar graph showed quantification of areas of positive cells. (G) The Arg-1 and CD206 protein levels at UUO 7d were analyzed by WB. β-actin was used as the loading control. Data were presented as means ± SEM. Each symbol in the bar graphs indicates an individual data value. *P* values were calculated using *t* test.

MST1/2 activation inhibitor (XMU-MP-1) was used to determine the role of MST1/2 in kidney fibrosis. Figure 8 A shows the UUO results on mice with or without XMU-MP-1; kidneys were collected 7 days after surgery. The fibrosis of these kidneys was detected by qRT-PCR and Sirius red staining. The mRNA expression of *Collage-I* and Sirius red staining demonstrated that the accumulation of collagen in the injury kidney of mice with XMU-MP-1 decreased compared with MST1/2 mice (without XMU-MP-1, Figure 8B-D). The Arg-1 positive area were decreased (Figure 8E, F). The Arg-1 and CD206 proteins also showed the same result (Figure 8G). Therefore, the inhibition of MST1/2 in macrophage reduced macrophage M2-type polarization *in vivo* and *in vitro*, and XMU-MP-1 impaired renal fibrosis.

## Discussion

As a component of innate immunity, macrophage plays a key role in inflammatory disease (such as bacterial infection), regulation of tissue repair (such as fibrosis), and various cancer 44, 45. Under LPS+IFN-γ or IL-4+IL-13 treatment, M0 macrophages can polarize into M1- or M2-type macrophage to play pro- or anti-inflammatory functions. M1 macrophage can secrete a large number of inflammatory factors, such as IL-1β, IL-6, and TNF-α to eliminate pathogenic microorganisms 46. M2 macrophage expresses Arg-1, Ym1, and Fizz1 and secretes anti-inflammatory factors for tissue repair 47. Therefore, how to regulate the polarization of M1 and M2 of macrophages and balance the pro- and anti-inflammatory levels are of great significance for the prevention and treatment of various inflammatory diseases. MST1/2 was initially reported as a key kinase of Hippo pathway in *Drosophila melanogaster*, and it regulates cell proliferation, apoptosis, and differentiation by inhibiting YAP nuclear translocation 48. MST1/2 deficiency increased the inflammatory response of macrophages in response to LPS or bacteria 19, 49. Our study provided evidence that MST1/2 plays a dual role in the process of macrophage polarization. We found upregulated MST1/2 expression and activated Hippo pathway under stimulation of LPS or IL-4+IL-13 (Figure 1G, H and I).

When XMU-MP-1 was used to inhibit MST1/2 activity, the RNA-Seq and qRT-PCR results showed that the expression of inflammatory cytokine under the stimulation of LPS and M2 marker decreased under the stimulation of IL-4+IL-13 (Figure 2). Therefore, the Hippo pathway plays a dual role in M1- and M2-type polarization. In particular, compared with WT mice, mice without MST1/2 exhibited more sensitive inflammatory response for LPS or bacterial infection, promoted macrophage polarization to M1, and enhanced the clearance rate to LPS-modified bacteria (Figure 4 and 5). Moreover, when mice were threatened with fibrosis induced by chronic inflammation, MST1/2 deficiency inhibited M2 polarization, and level fibrosis decreased (Figure 7 and 8). Thus, MST1/2 could be a crucial controller to adjust macrophage-related disease by regulating macrophage polarization, but the mechanism remains to be elucidated.

MST1/2 and Hippo pathway are associated with various inflammatory diseases 49-51. Multiple pathways regulate macrophage polarization, including the MAPK and NF-κB pathway 12. Zhao reported that the deletion of MST1 affected microglia activation, and then decreased stroke-induced brain injury via Src-MST1-IκB after cerebral ischemia-reperfusion injury 52. Li showed that STK4 in macrophage inhibited TLR4/9-induced inflammatory factor secretion, enhanced TLR3/4-triggeredd IFN-β production by binding to and degrading IRAK1, and similar results were obtained in inflammation-induced hepatocellular carcinoma 23. MST1 cKO in mouse embryonic fibroblasts and BMDM enhanced TNF-induced NF-κB target genes 53. Roh also found that MST1 ablation increased NF-κB-induced pro-inflammatory factor production, thus increasing the susceptibility of mice to LPS-induce tissue injury and septic shock 22. Therefore, MST1/2 regulates macrophage inflammation most likely through the TLR4 downstream NF-κB pathway. The RNA-Seq data showed similar results (Figure S2 A and B). The activation of NF-κB and classical TLR4 downstream pathway, namely, the MAPK pathway 42 were checked. As reported by Geng, MST1/2 deficiency did not affect LPS-induced phosphorylation of p38, ERK, and NF-κB p65 19 (Figure 3B). Next, the nuclear translocation of p65 was checked after LPS induce with or without MST1/2. YAP bonded with p65 to facilitate p65 nuclear translocation. The p65 location and the binding of p65 to YAP were checked. The WB, IF and Co-IP results showed that MST1/2 deficiency increased p65 accumulation in the nucleus by elevated YAP binding to p65 (Figure 3C-G and Figure S4 B, C). The results were same as Yang and Li reports 17, 23. The mechanism of MST1/2 deficiency promoted pro-inflammatory factor expression and M1 polarization by increasing the accumulation of p65 in the nucleus via YAP. The effect of MST1/2 inhibition on inflammatory microenvironment *in vivo* was determined using LPS-induce acute endotoxin shock model. The results were consistent with those reports in the literature and our results, in which MST1/2 deficiency increased lung inflammatory respond and injury.

Colistin is considered as the “last resort” antibiotic for the treatment of infections caused by extensively drug-resistant gram-negative bacteria 43, 54. mcr genes encode phosphoethanolamine (pEtN) transferases that modify the lipid A moieties of LPS in the outer membranes of gram-negative bacteria, thus reducing the net negative charge of LPS, and thereby lowering its affinity for colistin 55. mcr-1/3-modified LPS induced weaker macrophage response than native LPS *in vitro*, and *mcr-1/3* positive-* E. coli* and *A. Salmonicida* induced weaker macrophage response, causing bacterial accumulation in mice in response to the higher mortality rate compared with *mcr*-1/3 negative bacteria 24. Our data showed that mice with MST1/2 inhibition exhibited higher inflammatory response, clearance rate, and survival rate of mice than the *mcr3*-positive bacterial infection only (Figure 5E, G-I). Moreover, MST1/2 inhibition did not directly cause severe inflammation, and it could elevate the sensitive of inflammatory cell to bacteria to a limited extent. When bacterial infection or antimicrobial inflammatory response was already intense enough, the effect of MST1/2 inhibition on inflammation would be weakened. Therefore, MST1/2 plays a role in limiting the excessive inflammatory response in acute inflammatory infection. This feature of MST1/2 inhibition could be more suitable as a potential intervention mode for drug-resistant gram-negative bacteria, which induced weak macrophage response. The MST1/2 of macrophage plays different roles in acute inflammatory injury and chronic inflammation-induce tissue repair. At present, the best and most effective treatment for bacterial infection is antibiotic treatment 56. However, with the use of antibiotics, bacterial resistance has gradually become a complex problem 57. Thus, the regulatory role of MST1/2 in inflammatory diseases suggests that drugs that target MST1/2 should be developed to assist antibiotics in the treatment of macrophage-related inflammatory diseases, especially drug-resistant bacterial infections.

According to our present data, MST1/2 inhibited M2 polarization (Figure 2B, C, and G). KEGG analysis and previous study showed that the MEK/ERK and STAT6 pathways, which are regulated by IL-4+IL-13, are involved in the M2 polarization of macrophages 21 (Figure S2C). The activation of STAT6, MEK, and ERK was determined. WB results shows that MST1/2 cKO inhibited M2 polarization by decreasing the activation MEK/ERK and STAT6 pathway (Figure 6B). Previous report has shown YAP did not affect p-STAT6 in M2 polarization. Meanwhile, we found that inhibition of MST1/2 regulated M2 polarization by decreasing p-STAT6 independently of YAP and p-MEK/ERK dependently of YAP (Figure 6D-H). MST1/2 and YAP both could regulate M2 polarization, but mechanisms were different. The mechanism in which MST1/2 inhibition decreased the activation of MEK/ERK and STAT6 pathway was not determined, thus requiring further study. At least, MST1/2 inhibition could reduce the IL-4+IL-13-induced activation of STAT6 and MEK/ERK in macrophage independent and dependent of YAP, and MST1/2 inhibition impaired M2 polarization and renal fibrosis.

UUO is an important model to study fibrosis caused by chronic inflammation 31, 41. A variety of cells are involved in renal fibrosis of UUO mice model, such as epithelial cells, mesenchymal cells and macrophages, and MST1/2 is widely expressed in these cells. As reported in our previous and other study, MST1/2 deficiency in PDGFRα^+^ cells or tubule cells enhanced renal fibrosis 41, 58. M2-type macrophages play a role in creating a pro-fibrotic microenvironment by secreting TGF-β, PDGF, and TIMP to promote fibrosis 59, 60. Contrary to MST1/2 deficiency in PDGFRα^+^ cells or tubule cells, MST1/2 deficiency in macrophage inhibited M2 accumulation and fibrosis in the obstructed kidney of mice (Figure 7), revealing the role of MST1/2 in different cells on renal fibrosis. To determine the effect of MST1/2 inhibition on renal fibrosis, XMU-MP-1 was used to treat UUO mice. The results showed that renal fibrosis of mice treated with XMU-MP-1 was impaired (Figure 8). Therefore, in the early stage of fibrosis, XMU-MP-1 inhibited M2 accumulation in response to decreased pro-fibrotic microenvironment, thus reducing the transformation of fibroblast and epithelial cells into myofibroblasts and ECM release. The inhibitory effect was earlier and stronger than YAP activation in myofibroblasts caused myofibroblast proliferation. The underlying mechanisms will be explored in our subsequent studies.

XMU-MP-1 is a small molecule inhibitor that inhibits MST1/2 kinases activation, and its *in vivo* efficacy improved liver injury 27. In the present study, XMU-MP-1 reduced the bacterial load of tissues, and it enhanced inflammatory response and survival after mice with *mcr-3*-positive*-E.coli* infection (Figure 5E, G-I). This finding suggests the potential therapeutic effect of MST1/2 for immunosuppressive diseases. In renal fibrosis disease, XMU-MP-1 decreased renal fibrosis level (Figure 8). MST1/2 can be used as an inhibitor for research, as well as a small molecule drug for the treatment of certain macrophage-related inflammatory disease.

The main content of our article answers two questions: one is whether MST1/2 inactivation regulates macrophage polarization through YAP, and the other is whether MST1/2 inactivation affects macrophage polarization in inflammation and fibrosis processes. In summary, we found MST1/2 plays a dual role in macrophage polarization. MST1/2 deficiency in macrophage increased inflammatory M1 polarization by promoting nuclear accumulation of p65 to enhance inflammation via YAP mechanism. MST1/2 deficiency in macrophage decreased M2 polarization by reducing the STAT6 (independent of YAP) and MER-ERK (dependent of YAP) pathways to relieve fibrosis. Thus, MST1/2 is a key controller that regulates macrophage polarization and homeostasis. The identification of the role of MST1/2 and related pathway in macrophage activation provides potential targets for macrophage-directed disease diagnostic and therapeutic strategies. Moreover, MST1/2 inhibitor XMU-MP-1 could be used as a small molecule drug for the treatment of macrophage-related inflammatory disease.

## Supplementary Material

Supplementary figures.

## Abbreviations

MST1/2: mammalian sterile 20-like kinases 1 and 2
BMDM: bone marrow derived macrophage
UUO: unilateral uretera obstruction
NF-κB: nuclear factor kappa-light-chain-enhancer of activated B cells
STAT: signal transducer and activator of transcription
MCSF: macrophage-colony stimulating factor
VP: verteporfin
LPS: lipopolysaccharide
IFN: interferon
IL: interleukin
TNF: tumor necrosis factor
Arg: arginase
WB: western blot
IF: immunofluorescence
Co-IP: co-immunoprecipitation
RNA-Seq: RNA sequencing
cKO: conditional knockout
qRT-PCR: real time-polymerase chain reaction
