# Supplementary material for: Inactivation of MST1/2 Controls Macrophage Polarization to Affect Macrophage-Related Disease via YAP and Non-YAP Mechanisms
Source: Int J Biol Sci. 2024 Jan 12;20(3):1004–23. doi: 10.7150/ijbs.87057 (PMC10797691; doi:10.7150/ijbs.87057)
Supplement: Supplementary file 1 — Supplementary figures. [file ijbsv20p1004s1.pdf]

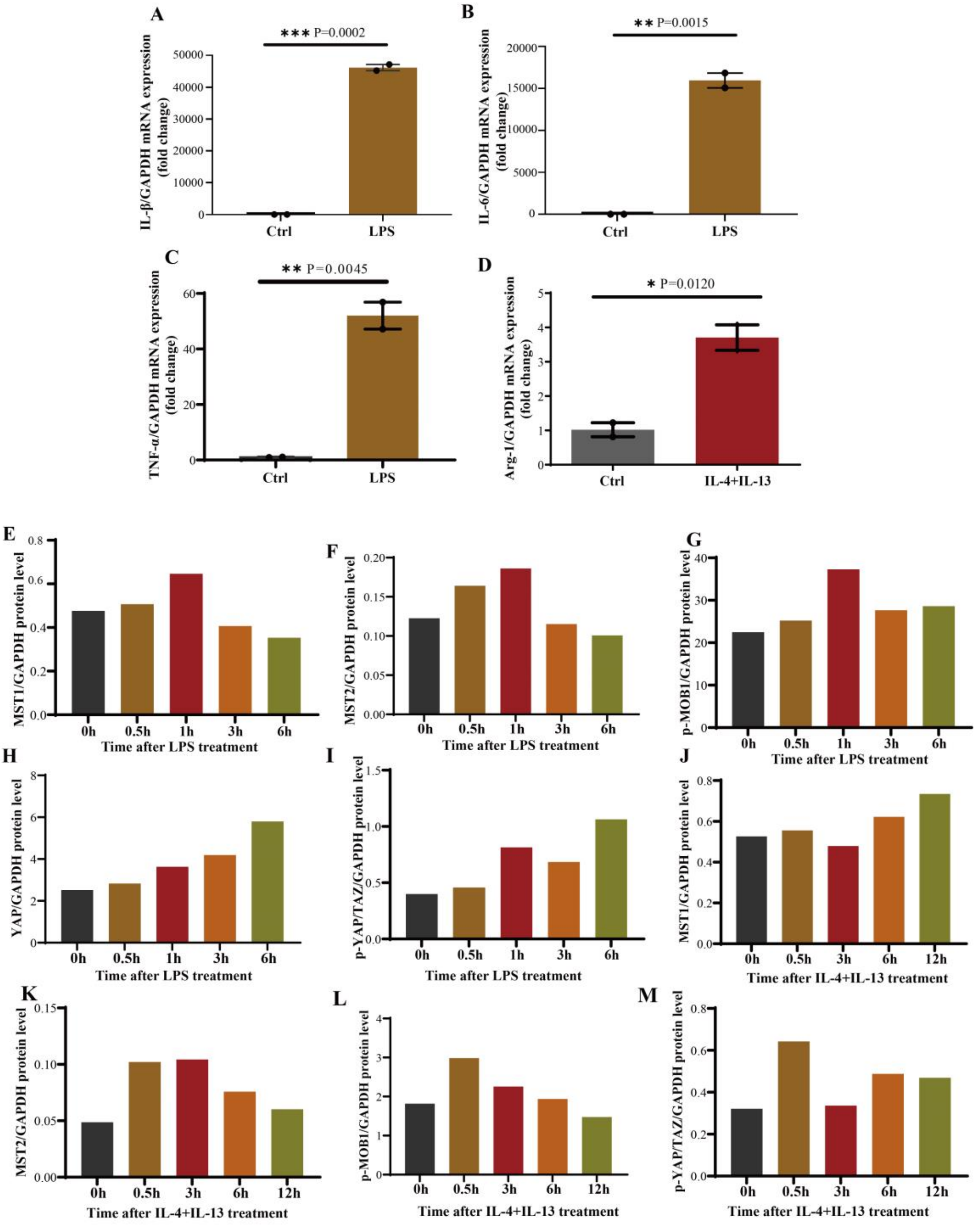

Figure S1 The mRNA and protein levels were analyzed. (A-D) The *IL-1β*(A), *IL-6*(B), *TNF-α*(C) and *Arg-1*(D) mRNA expression were analyzed after LPS or IL-4+IL-13 treatment. Data were presented as means ± SEM. *P* values were calculated using *t* test, n=2 fields per group. * compared to control group. (E-I) The MST1(E), MST2(F), p-MOB1(G), YAP (H) and p-YAP/TAZ (I) protein levels of RAW 264.7 cells were calculated after LPS treatment. (J-M) The MST1(J), MST2(K), p-

MOB1(L) and p-YAP/TAZ(M) protein levels of RAW 264.7 cells were calculated after IL-4+IL-13 treatment. GAPDH was used as the loading control.

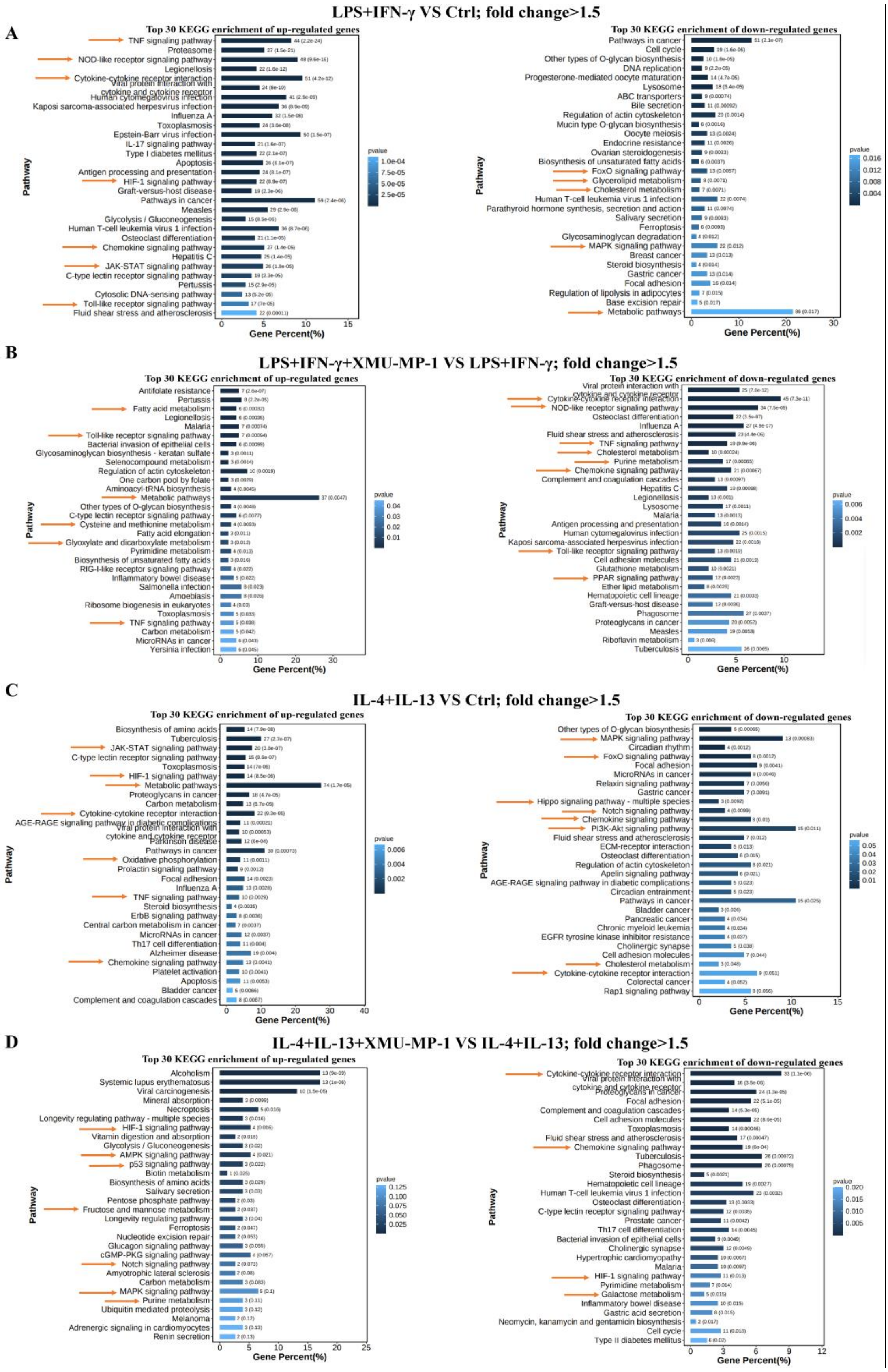

Figure S2 The KEGG enrichment of RNA-Seq data. (A-D) The KEGG enrichment of up-regulated and down-regulated genes of RNA-Seq (fold change > 1.5) compared LPS+IFN-γ to

control group (A), compared LPS+IFN-γ with XMU-MP-1 to LPS+IFN-γ (B), compared IL-4+IL-13 to control group (C) or compared IL-4+IL-13 with XMU-MP-1 to IL-4+IL-13 (D).

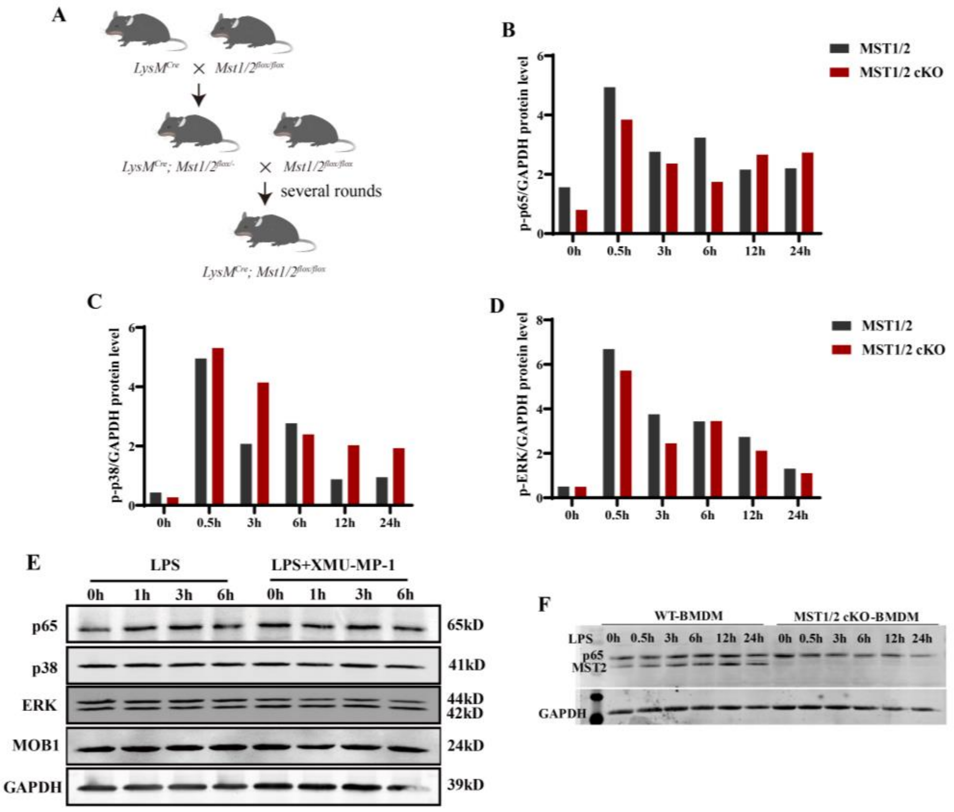

Figure S3 Mouse cross plot and protein levels were analyzed. (A) *LysM*^{Cre} mice were crossed with *Mst1/2*$^{flox/flox}$ mice to generate *LysM*^{Cre}*; Mst1/2*$^{flox/-}$ mice. Offspring mice were then hybridized with *Mst1/2*$^{flox/flox}$ mice to obtain *LysM*^{Cre}*; Mst1/2*$^{flox/flox}$. (B-D) The p-p65(B), p-p38(C) and p-ERK(D) protein levels of MST1/2- or MST1/2 cKO-BMDMs were calculated after LPS treatment. (E) The p65, p38, ERK and MOB1 protein levels of RAW 264.7 cells were analyzed by WB after LPS treatment for 0, 1, 3, and 6h. GAPDH was used as the loading control. (F) The p65 protein levels of MST1/2- and MST1/2 cKO-BMDMs were analyzed by WB after LPS treatment for 0, 0.5, 3, 6, 12 and 24h. GAPDH was used as the loading control.

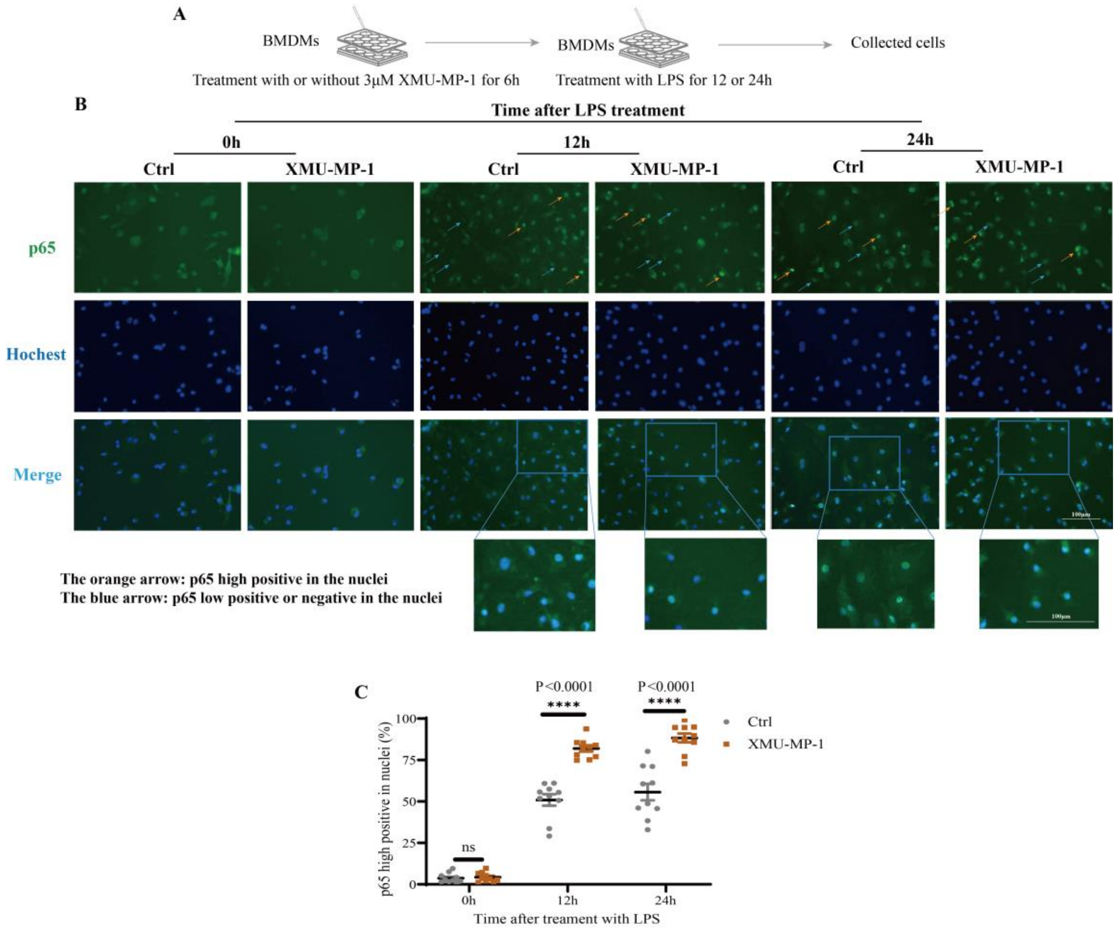

Figure S4 MST1/2 inhibition increased p65 accumulation in nucleus. (A) BMDMs were treated with or without 3μM XMU-MP-1 for 6h, and LPS induced for 12h or 24h. Then cells were collected for immunofluorescence. (B) The immunofluorescent staining of p65 in BMDMs with or without XMU-MP-1 was conducted. The orange arrow: p65 high positive in the nuclei. The blue arrow: p65 low positive or negative in the nuclei. Scale bars = 100 μm. (C) The p65 high positive in the nuclei (%) was calculated (p65 high positive in the nuclei (%) = the number of p65 high positive in the nuclei ×100 / the number of total nuclei). Data were presented as means ± SEM. *P* values were calculated using *t* test, n=10 fields per group. * compared to control group.

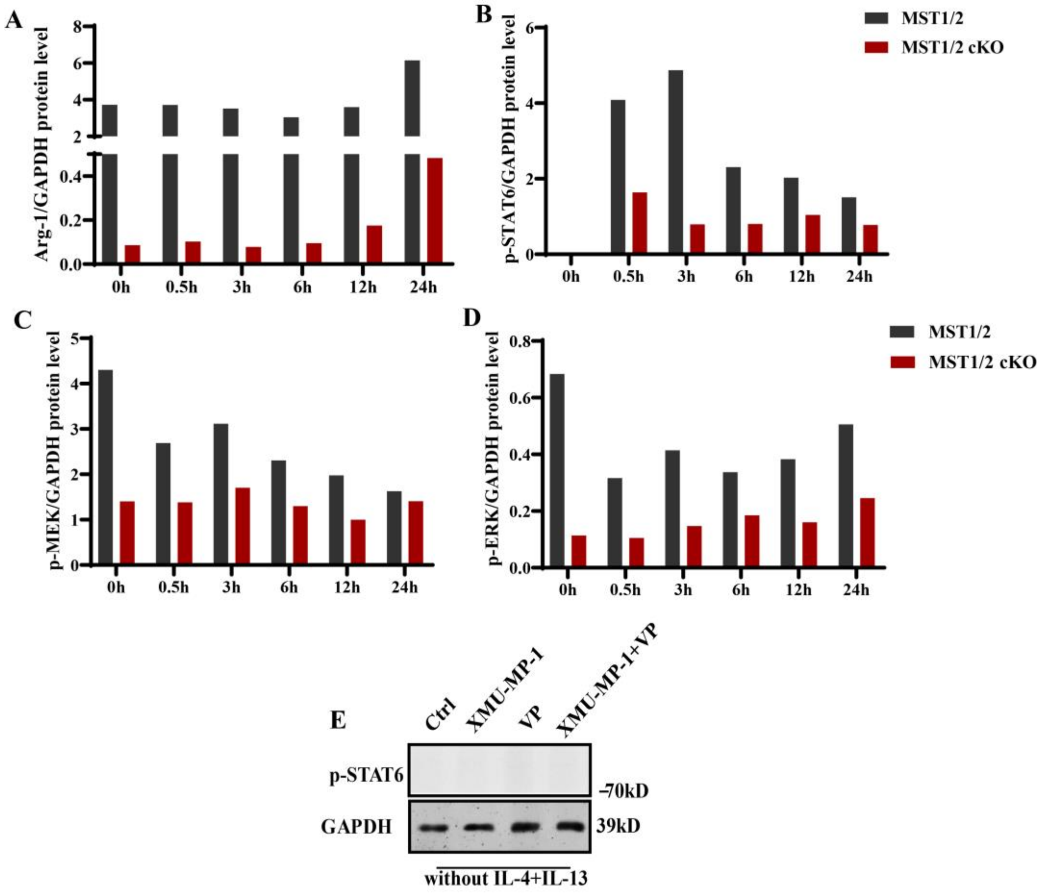

Figure S5 The protein levels were analyzed. (A-D) The Arg-1(A), p-STAT6(B), p-MEK(C) and p-ERK(D) protein levels of MST1/2-BMDMs or MST1/2 cKO-BMDMs were calculated after IL-4+IL-13 treatment. (E) The p-STAT6 protein level of RAW264.7 cells were analyzed by WB after XMU-MP-1 and/or VP without IL-4+IL-13.
